# Lactate-mediated histone lactylation promotes melanoma angiogenesis via IL-33/ST2 axis

**DOI:** 10.1038/s41419-025-08023-y

**Published:** 2025-10-06

**Authors:** Mao Zhao, Yuxuan Qian, Lin He, Taoxin Peng, Hanbin Wang, Xiangxu Wang, Linhan Jiang, Jinrong Fan, Hengxiang Zhang, Di Qu, Qing Zhu, Hao Wang, Shida Zhang, Chenyang Li, Xiwen Dong, Xianya Zhao, Huina Wang, Yuqi Yang, Xiuli Yi, Tao Zhao, Yu Liu, Jianglin Zhang, Guoqiang Zhang, Qiong Shi, Tianwen Gao, Chunying Li, Weinan Guo

**Affiliations:** 1 Department of Dermatology, Xijing Hospital, Fourth Military Medical University, Xi’an, Shaanxi China; 2 Department of Pathology and Institute of Molecular Pathology, The First Affiliated Hospital, Jiangxi Medical College, Nanchang University, Nanchang, Jiangxi China; 3 Department of Oncology, Xijing Hospital, Fourth Military Medical University, Xi’an, Shaanxi China; 4 Department of Dermatology, Air Force Medical Center, PLA, Beijing, China; 5 Department of Dermatology, Shenzhen People’s Hospital (The Second Clinical Medical College, Jinan University, The First Affiliated Hospital, Southern University of Science and Technology), Shenzhen, Guangdong China; 6 Department of Dermatology, The First Hospital of Hebei Medical University, Shijiazhuang, Hebei China

**Keywords:** Melanoma, Cancer

## Abstract

The pathogenesis of cancer is complicated, with metabolic reprogramming and angiogenesis as the hallmark characteristics. Recent reports have unveiled that the glycolytic metabolite lactate could modify histone lactylation to epigenetically regulate gene expressions and biological processes in cancer, while the effect on tumor angiogenesis remains elusive. By taking advantage of melanoma as the model, we first proved that lactate and histone lactylation facilitated melanoma angiogenesis both in vitro and in vivo. Then, through RNA-sequencing and a series of biochemical assays, we found that lactate promoted the transcription of suppression of tumorigenicity 2 (ST2) in tumor-associated endothelial cells via the enhancement of histone lactylation at its promoter, so that to increase the response of endothelial cells to pro-angiogenic interleukin-33 (IL-33) stimulation. In addition, lactate could also suppress high endothelial venules transition of endothelial cells, which was critical for tumor development. Ultimately, the effect of anti-angiogenic drug synergized with lactate dehydrogenase (LDH) inhibition/ST2 inhibition on melanoma growth was proved in vivo. Taken together, we demonstrated that lactate-mediated histone lactylation promotes melanoma angiogenesis via IL-33/ST2 axis, which delineated a novel regulatory relationship among lactate, histone lactylation and angiogenesis in cancer, and provided a promising combined therapeutic strategy to target angiogenesis from the perspective of cell metabolism and epigenetics in cancer.

## Introduction

Melanoma, a highly aggressive skin cancer that originates from epidermal melanocytes, is well-known for its tendency to proliferate and metastasize uncontrollably [1]. A combination of environmental and intrinsic factors can influence the development of melanoma, with hallmark characteristics including metabolic reprogramming, immune evasion, and angiogenesis [2]. Currently available treatment methods for patients with melanoma include immune checkpoint inhibitors to combat immune evasion, targeted therapies for genetic mutations, and anti-angiogenic agents to inhibit tumor vascularization [3]. Despite these therapeutic advances, attaining long-term survival in patients with advanced melanoma remains a persistent and multifaceted challenge [1], highlighting the necessity for further investigation of the mechanisms to identify alternative potential therapeutic strategies.

Angiogenesis is a key driver of melanoma progression, as evidenced by the observation that melanoma cells exhibit significantly higher expression of vascular endothelial growth factor (VEGF) and VEGF receptor (VEGFR) expression than normal melanocytes [4]. Increased angiogenesis promotes melanoma cell survival by facilitating oxygen and nutrients delivery while simultaneously fostering immune cell infiltration and function. This process involves the recruitment of immunosuppressive cells and modulation of immune checkpoint molecule expression [5]. Based on the multifaceted role of angiogenesis in tumor development, anti-angiogenic therapy has emerged as a promising strategy in cancer treatment, and the most broadly utilized anti-angiogenic drugs are antibodies and tyrosine kinase inhibitors (TKIs) that target the VEGF-VEGFR pathway [6]. Although the therapeutic effect of anti-angiogenic agents such as bevacizumab as monotherapy for melanoma remains limited [7], the combination of anti-angiogenic drugs with immunotherapy in melanoma treatment displays more prominent therapeutic efficacy. For instance, the combination of atezolizumab and bevacizumab displayed significant therapeutic benefits and a favorable safety profile in patients with advanced mucosal melanoma [8]. Despite these advances, some factors might also render the drug resistance and compromise treatment efficacy. These include compensatory upregulation of alternative angiogenic factors and a hypoxic, lactate-rich tumor microenvironment [9]. Consequently, further elucidation of the mechanisms underlying melanoma angiogenesis is imperative for the development of potential treatment choices.

The Warburg effect, a fundamental metabolic hallmark of cancer, indicates tumor cells undergoing aerobic glycolysis even with sufficient oxygen, resulting in substantial lactate accumulation [10, 11]. Lactate has evolved beyond its conventional identity as a metabolic byproduct [12], intracellular signaling pathway transduction [13], and immune response modulation [14]. Of note, previous reports have revealed that lactate exerts a critical function in facilitating tumor angiogenesis [15]. In particular, endothelial cells possess the capacity to uptake lactate generated by tumor cells, which activates hypoxia-inducible factor 1 (HIF-1) to increase VEGF expression and promote angiogenesis [16]. Additionally, lactate can promote angiogenesis by functioning as an agonist of G protein-coupled receptor 81 (GPR81) in endothelial cells [15]. Recent studies have uncovered that lactate mediates protein lactylation, a post-translational modification involving lactyl-group conjugation to specific amino acid residues, such as lysine [17]. Lactylation not only directly affects proteins by influencing their structure, expression, and function, but also regulates gene transcription by modulating histones, thereby impacting cellular behavior [18]. In microglia, increased lactylation of Yin Yang-1 (YY1) facilitates angiogenesis by transcriptionally activating Fibroblast Growth Factor (FGF) [19]. In vascular endothelial cells, histone H3 lysine 9 lactylation (H3K9la) modulates VEGF-induced angiogenesis through histone deacetylase 2 (HDAC2) -mediated feedback loop [20]. While previous reports established the fundamental role of lactylation in the regulation of angiogenesis, the precise mechanisms governing lactate-mediated tumor angiogenesis—particularly through histone lactylation-dependent epigenetic regulation—require further elucidation.

Beyond angiogenesis driven primarily by endothelial cell proliferation, endothelial cells also shape the tumor microenvironment through the formation of high endothelial venules (HEVs). HEVs are specialized blood vessels in the peripheral vasculature that mediate lymphocytes to lymph nodes and other secondary lymphoid organs [21], which can function as a major route for peripheral blood lymphocytes to enter tumors. HEV density demonstrates a strong correlation with T and B cells’ infiltration in tumor microenvironment, and therapeutic induction of HEVs has been shown to promote the effectiveness of immunotherapy by facilitating the entry of lymphocytes into tumor beds [22, 23]. However, research on HEV regulation in cancer remains nascent, with no existing studies investigating potential lactate-mediated effects on tumor-associated HEVs.

With growing recognition of lactate’s multifaceted role in the tumor microenvironment—particularly its epigenetic regulation of gene expression through histone lactylation—this study investigates the mechanistic basis by which lactate and histone lactylation regulate tumor angiogenesis and HEV formation in melanoma. After having systematically characterized the effect of lactate and histone lactylation on melanoma angiogenesis both in vitro and in vivo, followed by mechanistic exploration through RNA-sequencing and biochemical validation, we further examined the potential influence of lactate and histone lactylation on HEV development. Finally, we evaluated the therapeutic potential of combining LDH inhibition with anti-angiogenic therapy.

## Methods

### Cell culture and reagents

The human cutaneous melanoma cell line A2058, A375 and murine melanoma cell line B16F10 were obtained from American Type Culture Collection. Human umbilical vein endothelial cells (HUVEC) were purchased from SAIOS (China). All cell lines used in this study were authenticated within the last three years using short tandem repeat (STR) profiling and confirmed negative for mycoplasma contamination. The melanoma cell lines were cultured in DMEM (G4511-500ML, Servicebio, China) with 10% (v/v) fetal bovine serum (FBS, 164210, Procell, China) and 1% (v/v) penicillin/streptomycin (V900929100, Sigma-Aldrich, USA). Reagents used in this article were sodium lactate (71718, Sigma-Aldrich, USA), A-485 (S8740, Selleck, USA), IL-33 (HY-P7041, MCE, China), Mitomycin C (HY-13316, MCE, China), Lactate Dehydrogenase A (LDHA) inhibitor FX-11 (GC34011, GLPBIO, USA), Puromycin, Sterile (GC16384, GLPBIO, USA), Tribromoethanol (M2910, Nanjing Aibei Biotechnology Co., China).

### Mice model and treatments

The animals involved in experiment were conducted in accordance with standards approved by the Institutional Animal Care and Use Committee at Air Force Medical University. All C57BL/6 mice utilized in the experimental procedures were female, aged 8 weeks, and obtained from Air Force Medical University Laboratory Animal Center, China. All animal experiments utilized C57BL/6 mice exclusively. Prior to any experimental procedures, mice were randomly assigned to pre-defined control and experimental groups. All subsequent procedures were conducted according to this predetermined group allocation. Seven days before the initiation of the experiment, all C57BL/6 mice were administered a subcutaneous injection of B16F10 cells (5 × 10^5^). Tumor dimensions were quantified using a caliper, ensuring that the maximum diameter did not exceed 2 cm. For treatments, FX-11 (2.5 mg/kg), NaHCO3 nanoparticles (20 mg/kg), IL-33 (10 μg/kg), A-485 (2.5 mg/kg), DC101 (10 mg/kg), and anti-ST2 antibody (1 mg/kg) were injected intraperitoneally or intra-tumoral every other day for 10 days. Following euthanasia, tumor tissues were excised and divided for subsequent analyses. One portion was fixed overnight with 4% paraformaldehyde to facilitate subsequent immunofluorescence staining, while the rest of tumor tissue was conducted into single-cell suspension for flow cytometric analysis.

### The establishment of melanoma-associated endothelial cells (TA-HUVEC) via co-culture system

A2058 /A375 and HUVEC cells were co-cultured by using transwell inserts with a 3 μm pore filter (3402, Falcon). A2058 cells (1 × 10^5^ cells/mL) were resuspended in 1 mL DMEM and seeded into the upper chamber, while HUVECs were resuspended at the same density in 2 mL of DMEM and seeded in the lower chamber. Then co-cultured for 72 h at 37 °C, 5% CO_2_ to generate tumor-associated HUVECs (TA-HUVECs). A2058 cell supernatant (supernatant: DMEM = 3:1) was used to culture TA-HUVEC afterwards.

### CCK8 assay

Cells (5 × 10^3^ /well) were seeded into a 96-well culture plate. Cell viability was subsequently assessed at 0, 24, 48, and 72 h intervals by the CCK8 assay (C0038, Beyotime Biotechnology, China), following the standardized protocol provided by the manufacturer. At the end of indicated treatment, the supernatant was aspirated, and 200 μL/well of CCK-8 working solution was dispensed into the plate. Then incubated for 2 h at 37 °C, the absorbance of the solution was quantified at 450 nm wavelength, and the corresponding optical density (OD) values were tested.

### Transwell migration assay

Transwell inserts equipped with an 8 μm pore filter (Falcon, USA) were used. Cells (4 × 10^4^cells/mL) were resuspended in 200 μL of serum-free DMEM supplemented with mitomycin C (5 μg/mL, HY-13316, MCE, China) then seeded into both upper and lower chamber, while 600 μL of medium containing 10% FBS was added to the lower chamber as a chemoattractant. Then incubated for 48 h to facilitate migration. Cells on the upper surface of the membrane were carefully removed, the migrated cells on the lower membrane were fixed with 4% paraformaldehyde and stained by a 1:100 dilution of crystal violet (C0121, Beyotime Biotechnology, China) for 10 min. The membrane was captured by microscope (Nikon-Eclipse, Japan) with 4× magnification. ImageJ (version 1.8.0; USA) was used to quantify the cells migrating into the lower chambers.

### Wound healing assay

TA-HUVEC and HUVEC cells were seeded in 6-well plates (6 × 10^5^ cells per well) and cultured until they reached 90% confluency. A linear wound was introduced into the well using a steriled 200 μL pipette tip. The plate was gently rinsed 3 times with PBS, then 3 mL serum-free medium supplemented with mitomycin C (5 μg/mL, HY-13316, MCE, China) was added to suppress cell proliferation. The cells were then incubated for 24 to 48 h. Wound closure was monitored and photographed by inverted microscope (Nikon Eclipse, Japan) at 4× magnification. The migration area was quantitatively analyzed using ImageJ (version 1.8.0, USA) to determine the extent of cell migration into the scratched region.

### Tube formation assay

Matrigel (0827245, ABW, China) was thawed and dispensed into 96-well plates (50 μl/well) and polymerized at 37 °C for 1 h. HUVEC or TA-HUVECs cells that are in good condition for 3-5 passages and allow to be 80% confluent within 24 h. For serum starvation, the supernatant was replaced with DMEM without FBS (200 μl/well), followed by 24 h of culture. Subsequently, HUVECs or TA-HUVECs (3 × 10^4^) were seeded onto the Matrigel layer, then incubated at 37 °C in 5% CO_2_, and photographed at 6 h by inverted microscope (Nikon-Eclipse, Japan) with (40×) magnification.

### Western blot

Cells were washed and subsequently lysed in RIPA buffer (P0013B, Beyotime, Shanghai, China) supplemented with a protease inhibitor cocktail (5871, CST, USA). The lysates were incubated on ice to ensure complete lysis and then centrifuged at 12,000 × *g* for 10 min. The protein concentration was determined by bicinchoninic acid (BCA) protein assay kit (A55860, Thermo Fisher Scientific, USA). Twenty microgram protein was applied. After electrophoresis, and placed onto PVDF membranes and blocked with 5% non-fat milk prepared in 0.1% TBST for 1 h at room temperature. Then incubated at 4 °C overnight with primary antibodies: CD31 (1:5000, 11265-1-AP, Proteintech, China), VEGFA (1:1000, ab39638, Abcam, UK), H3K18la (1:1000, PTM-1406RM, PTM BIO, China), ST2(1:1000, ZRT1338, Merck, Germany), PNAd (1:1000, ab111710, Abcam, UK), ICAM1(1:1000, ab53013, Abcam, UK), Phospho-Akt(1:2000, 4060T, Cell Signaling Technology, USA), Akt(1:1000, 4691T, Cell Signaling Technology, USA), β-tubulin (1:5000, 66240-1-Ig, Proteintech, China) and GAPDH (1:1000, ab8245, Abcam, UK). Then washed with TBST and incubated with HRP-conjugated secondary antibodies (Cell Signaling Technology, USA) for 1 h at room temperature. Then detected by enhanced chemiluminescence western blot detection kit (Beyotime Biotechnology, China) and imaged with a ChemiDoc XRS+ System (Bio-Rad, USA).

### Quantitative RT-PCR (qRT-PCR)

The quantification of RNA transcript expression levels was performed using qPCR. RNA integrity was verified through lab-on-chip technology, following the manufacturer’s guidelines, and spectrophotometric assessment. Total RNA was isolated from HUVECs utilizing TRIzol (Thermo Fisher Scientific, USA), adhering to the standard RNA extraction protocol. Subsequently, 1.5 mg of the total RNA was reverse-transcribed into cDNA using reverse transcriptase PCR. The cDNA was then subjected to amplification via a rtPCR system (Bio-Rad, USA) with SYBR-Green PCR Master Mix. Beta-actin served as the internal control for normalization. All primer sequences are detailed and were procured from Tsingke Biotechnology (China) in Supplementary Table 1.

### RNA-sequencing technology

The integrity of RNA was evaluated utilizing the RNA Nano 6000 Assay Kit on the Bioanalyzer 2100 system (Agilent Technologies, USA). Total RNA served as the initial material for RNA library preparation. cDNA fragments were selectively amplified using the AMPure XP system. Index-coded samples were clustered on a cBot Cluster Generation System with the TruSeq PE Cluster Kit v3-cBot-HS (Illumina). Post-cluster generation, the prepared libraries were sequenced on an Illumina Novaseq platform, generating 150 base-pair (bp) paired-end reads.

### Chromatin immunoprecipitation (ChIP)

ChIP assay was conducted using lysates derived from HUVEC cells subjected to specified treatments by Chromatin IP Kit (Catalog No. 56383, Cell Signaling Technology, USA). Initially, cellular samples were cross-linked with 1% formaldehyde for a duration of 20 min, followed by quenching with glycine for 5. Then harvest and enzymatically digest with micrococcal nuclease for 20 min. The enzymatic reaction was terminated using EDTA, after which the DNA was sheared via sonication. Immunoprecipitation was then performed using an anti-H3K18la antibody (Catalog No. PTM-1427RM, PTM BIO, China). The immunoprecipitated DNA underwent treatment by RNase A and proteinase K, purified through phenol-chloroform extraction and ethanol precipitation. Both the immunoprecipitated DNA and input DNA were subsequently quantified using qPCR analysis.

### Dual-luciferase reporter assay

The 2000-bp promoter region of ST2 was chemically synthesized by Tsingke Biotechnology (Beijing, China) and subsequently cloned upstream of the firefly luciferase gene in the pGL3-Basic vector (Promega, USA) for reporter gene assays. TA-HUVEC cells were seeded in 6-well plates and co-transfected with 1 μg of the promoter-reporter construct and 50 ng of the pRL-TK Renilla luciferase control vector (Promega, USA) using 5 μL Lipofectamine 3000 (Invitrogen, USA). At 24 h post-transfection, cells were treated for 24 h with either 10 mM NALA, 10 mM NALA plus 10 μM A485, or vehicle control. Luciferase activity was then measured using the Dual-Luciferase Reporter Assay System (Promega, USA) according to the manufacturer’s instructions, with firefly luciferase activity normalized to Renilla luciferase activity for each sample. All experiments were performed in three independent biological replicates with triplicate technical repeats.

### L-Lactic acid colorimetric assay

L-Lactic Acid levels were analyzed by Lactic Acid assay kit (NanJing Jiancheng Bioengineering, A019-2-1, China). Samples were mixed with enzyme solution, chromogen by step, and incubated at 37 °C for 10 min. Then add terminator to stop reaction. A wavelength of 530 nm was used with a 1 cm optical path for colorimetry.

### Flow cytometry analysis

For flow cytometric analysis, tissues were mechanically dissociated by syringe plunger and 70 µm strainer (352350, Corning, USA) to obtain single-cell suspensions. The isolated cells were resuspended in cold PBS supplemented with 10% FBS (164210, Pricella, China). Viability staining was performed using Zombie UV dye (423108, Biolegend, USA) to distinguish live and dead cells. Then blocked with Fc blocking antibody (14-9161-73, Invitrogen, USA) for 15 min. Following blocking, cells were washed and subjected to surface staining with fluorochrome-conjugated antibodies: Pacific Blue anti-mouse CD45 (1:1000 dilution, 103126, Biolegend, USA), APC anti-mouse CD31 (PECAM-1) antibody (1:1000 dilution, 160210, Biolegend, USA), and Alexa Fluor 488 anti-mouse MECA-79 (1:1000 dilution, 53-6036-80, eBioscience, USA). After staining, cells were washed, centrifuged, and fixed by 200 μL of a fixation/permeabilization solution (00-5523-00, Invitrogen, USA). Analysis was performed by BD LSRFortessa™ Flow Cytometer (BD Biosciences, USA), analyzed by FlowJo software (Version 10.10.0, USA), and GraphPad Prism (Version 9.0, USA).

### Immunofluorescence staining analysis

The expression levels of CD31 and MECA-79 in HUVEC/TA-HUVEC cells, as well as CD31, MECA-79, H3K18la, and ST2 in tumor specimens, were assessed using immunofluorescence staining. HUVEC/TA-HUVEC cells (1 × 10^5^) were seeded in glass-bottom dishes (706201, NEST, China) and cultured for 48 h. The cells were fixed and washed three times. For tumor tissue analysis, murine melanoma samples were harvested post-sacrifice and fixed, then sectioned into 5-µm-thick slices. Antigen retrieval was performed followed by permeabilization with 0.5% Triton-X-100 for 15 min and blocked with goat serum (16210064, Thermo Fisher Scientific, USA). Primary antibodies, including anti-CD31 (1:400, 11265-1-AP, Proteintech, China), anti-H3K18la (1:400, PTM-1406RM, PTM BIO, China), anti-ST2 (1:400, ab25877, Abcam, UK), and anti-MECA-79 (1:400, SC-19602, Santa Cruz Biotechnology, USA), were applied and incubated at 4 °C overnight. Then the samples were incubated with secondary antibodies: Cy3 (1:1000, ab6939, Abcam, UK), Alexa Fluor 488 (1:1000, ab150077, Abcam, UK), and DAPI (1:1000, ab285390, Abcam, UK). Fluorescence imaging was conducted by laser scanning confocal microscope (LSM 980, Zeiss, Germany), and analysis of fluorescence was performed by ImageJ(version 1.8.0, USA).

### Immunohistochemistry (IHC)

Melanoma tissues were paraffin-embedded and sectioned at 4 μm. Tissue sections and tumor tissue microarray (TMA MME1004j, Taibsbio, China) were blocked with SP Rabbit and Mouse HRP Kit (CW2069S, CWBIO, China) for 1 h, then incubated at 4 °C overnight with the following primary antibodies then incubated with CD31 antibody(1:200, 11265-1-AP, Proteintech, China) and H3K18la antibody(1:400, PTM-1406RM, PTM BIO, China), VEGFA antibody (1:1000, ab39638, Abcam, UK) and ST2 antibody (1:400, ab25877, Abcam, UK). Immunohistochemical (IHC) staining was developed using AEC Peroxidase Substrate Kit (A1020, Solarbio, China).

### Microvascular imaging of living tumors

Mice are anesthetized with tribromoethanol (20 ml/kg, M2910, Nanjing Aibei Biotechnology, China), and hair was removed by depilatory cream. For microvascular imaging, animal OCT vascular imaging system (model: ISOCTA, UK) was used for tumors microvascular photographing and microvascular morphology processing. Vascular parameters (diameter and cross-sectional area) were quantified from 4D-OCT volumetric scans (XYZT coordinates) using a semi-automated segmentation algorithm. Briefly, OCTA (optical coherence tomography angiography) images were generated by analyzing intensity-based decorrelation signals between consecutive B-scans to highlight blood flow. Vessels were segmented using a Hessian filter-based approach to enhance tubular structures, followed by adaptive thresholding to isolate vascular lumens. Vessel diameters were measured as the full-width at half-maximum (FWHM) of intensity profiles perpendicular to the vessel axis, while cross-sectional areas were calculated from binary masks of segmented lumens. Only vessels with diameters >15 μm (system resolution limit) were included. All analyses were performed using custom MATLAB scripts (MathWorks).

### Statistical analysis

Using GraphPad Prism software (version 9.0, USA), data are expressed as mean ± standard deviation (SD) and were obtained from at least three independent experimental replicates. For comparisons between two groups, an unpaired Student’s *t*-test was employed. For comparisons involving multiple groups, one-way analysis of variance (ANOVA) was performed. And Tukey’s post-hoc test for multiple comparisons. A *P* value of < 0.05 was considered statistically significant.

## Results

### The establishment and validation of melanoma-associated endothelial cells

**Fig. 1:**
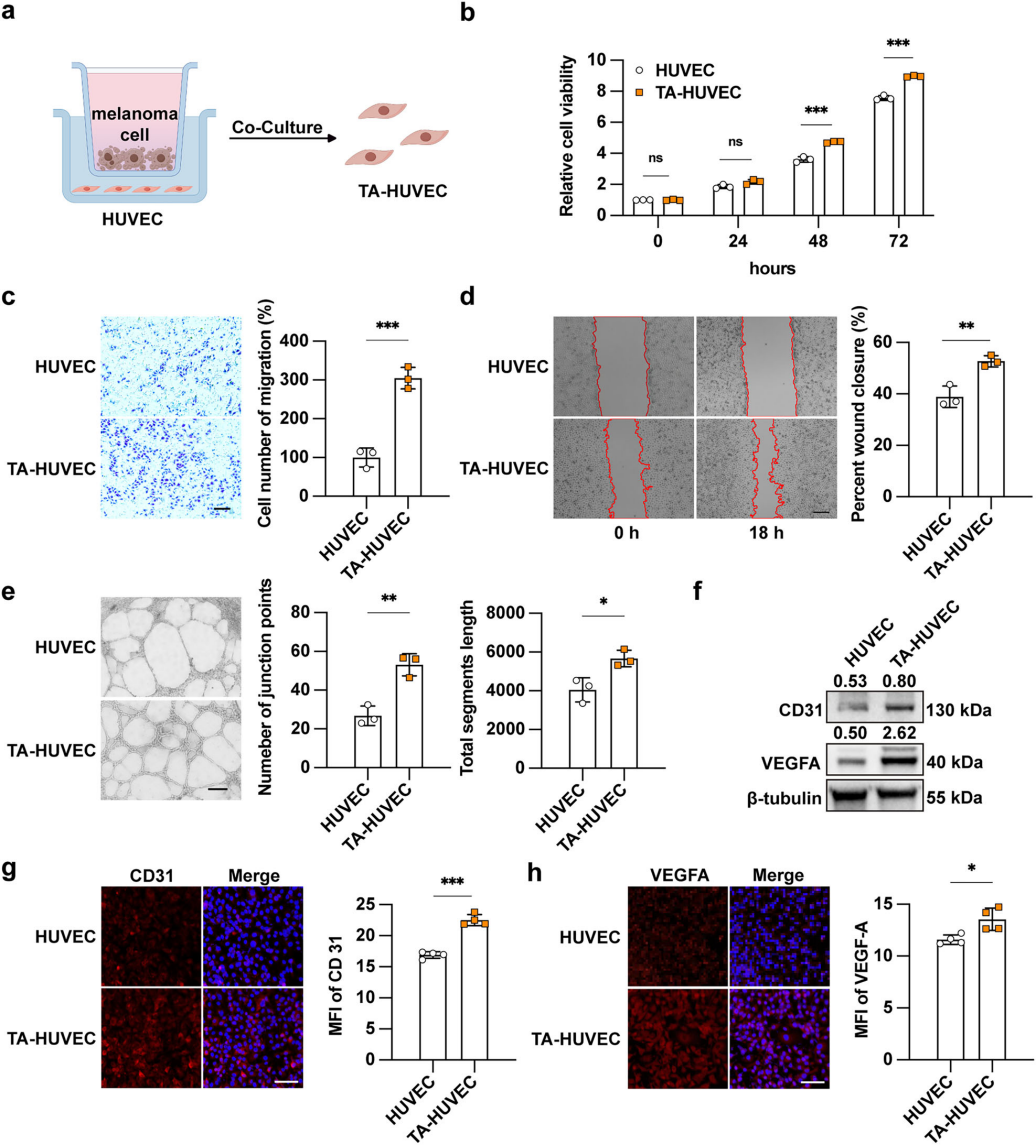
The establishment and validation of melanoma-associated endothelial cells. **a** Schematic view of TA-HUVEC induction. Transwell chamber is used to co-culture HUVECs with human melanoma cell line A2058 for 72 h to establish TA-HUVECs. **b** Cell viability of HUVEC and TA-HUVEC using CCK8 assays at 0, 24, 48, 72 h. **c**, **d** Cell migration of HUVEC and TA-HUVEC using transwell and wound-healing assay. Scale bar, 200 μm. **e** Tube formation of HUVEC and TA-HUVEC. Scale bar, 200 μm. **f** Immunoblotting analysis of CD31 and VEGFA expression in HUVEC and TA-HUVEC. **g**, **h** Immunofluorescence staining of CD31 and VEGFA in HUVEC and TA-HUVEC. Scale bar, 100 μm. *P* value was calculated by unpaired Student’s *t*-test, mean ± SEM, *P < 0.05, **P < 0.01, ***P < 0.001.

To investigate the pro-angiogenic effect of melanoma cell-derived lactate, we firstly establish melanoma-associated endothelial cells (TA-HUVEC) by co-culture of HUVEC and human melanoma cell line A2058, so that to induce the formation of TA-HUVEC (Fig. 1a). Compared to HUVEC, the proliferative capacity of TA-HUVEC exhibited a significant increase at different time points (Fig. 1b). Both the transwell assay and wound-healing assay revealed markedly increased migratory potential of TA-HUVEC in comparison to HUVEC (Fig. 1c, d). What’s more, the tube formation assay demonstrated that TA-HUVEC had superior angiogenic capability that HUVEC, as evidenced by significant increase in junction points and total segments length (Fig. 1e). These findings collectively demonstrated that melanoma cells could facilitate angiogenesis without direct endothelial contact. The concurrent western blot analysis revealed that both the expressions of CD31 and Vascular Endothelial Growth Factor A (VEGFA), which are markers of endothelial cells, were significantly elevated in TA-HUVEC compared to HUVEC (Fig. 1f). Consistent with these findings, immunofluorescence staining analysis also revealed markedly enhanced signal intensity of CD31 and VEGFA in TA-HUVEC (Fig. 1g, h). To validate these observations, we used another melanoma cell line A375 to co-culture with HUVEC and establish TA-HUVEC. Similarly, the proliferative capacity of TA-HUVEC was prominently increased compared with HUVEC (Supplementary Fig. S1a). Moreover, immunoblotting analysis demonstrated that the expressions of both CD31 and VEGFA were potentiated in TA-HUVEC, reinforcing the facilitative role of melanoma cells in endothelial cells proliferation (Supplementary Fig. S1b). Based on these consistent findings, we use A2058-derived TA-HUVEC (co-culture with A2058 cell line) as a representative model that could mimic the characteristic of melanoma-associated endothelial cells for subsequent investigations.

### Lactate and histone lactylation promote angiogenesis

**Fig. 2:**
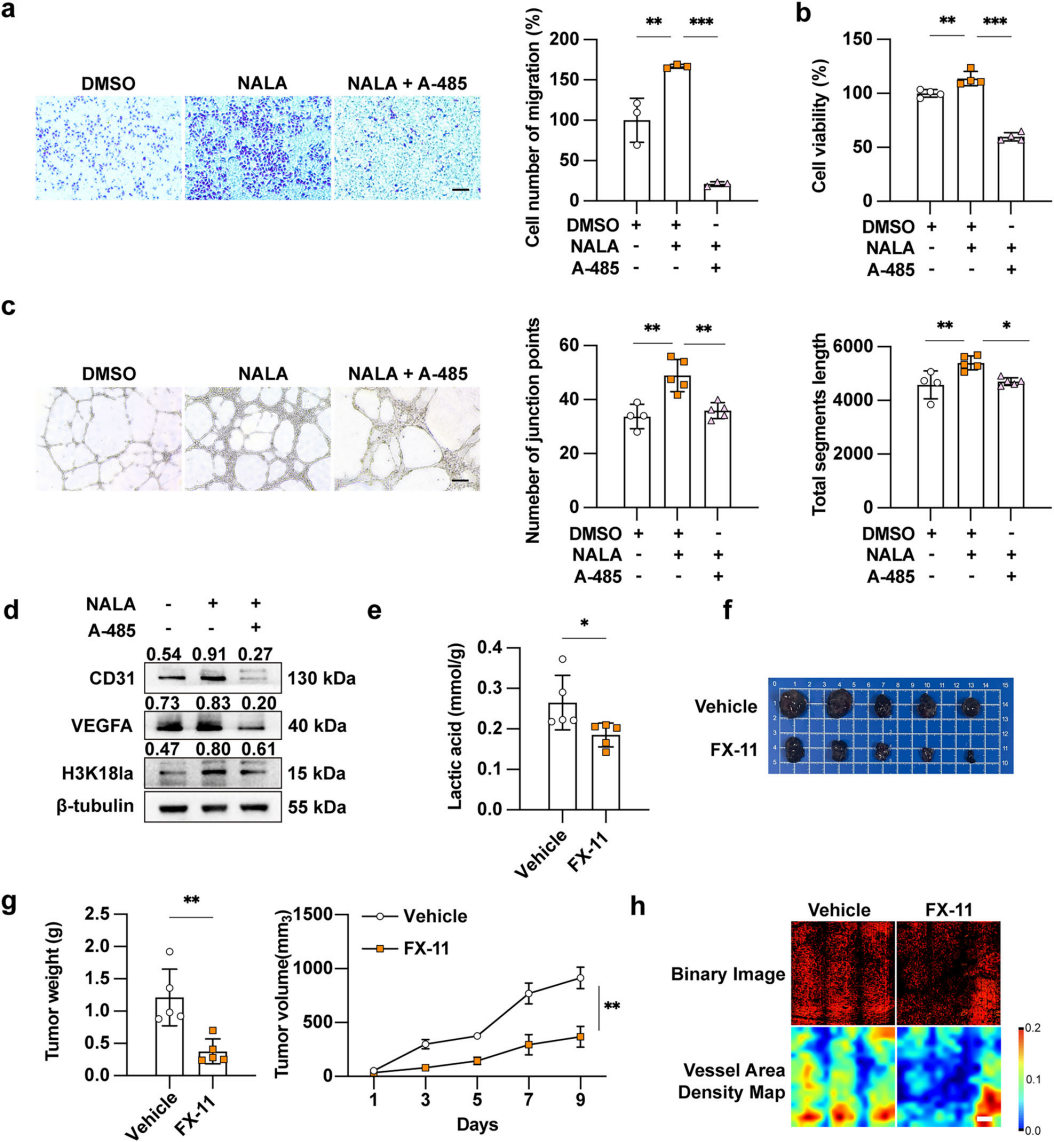
Lactate and histone lactylation promotes angiogenesis. **a** Cell migration of TA-HUVEC treated with NALA (10 mM) and/or A-485 (10 μM) for 48 h. Scale bar, 200 μm. **b** Cell viability of TA-HUVEC treated with NALA (10 mM) and/or A-485 (10 μM) for 48 h. **c** Tube formation of TA-HUVEC treated with NALA (10 mM) and/or A-485 (10 μM) for 48 h. Scale bar, 200 μm. **d** Immunoblotting analysis of H3K18la, CD31 and VEGFA expression in TA-HUVEC treated with NALA (10 mM) and/or A-485 (10 μM) for 48 h. **e** Tumor lactic acid content in each group (mmol/g protein). **f** Images of B16F10 melanoma cell xenografts with or without FX-11 treatment isolated from C57BL/6 mice. **g** Tumor weights and volumes in each group (*n* = 5). **h** 4D-OCT intravital imaging of microvasculature in tumor with indicated treatments (*n* = 5, Scale bar, 300 μm). *P* value were calculated using an unpaired Student’s *t*-test for comparisons between two groups, and were calculated using one-way ANOVA followed by Tukey’s multiple comparisons test for comparisons more than two groups. Mean ± SEM, *P < 0.05, **P < 0.01, ***P < 0.001.

After the establishment of TA-HUVEC, we next determine whether tumor cell-derived lactate and its-dependent histone lactylation could impact angiogenesis in melanoma. We treated TA-HUVEC with exogenous sodium lactate (NALA) in combination with A-485, a selective inhibitor of the histone lactytransferase P300 [17]. As a result, NALA treatment could facilitate cell proliferation and migration of TA-HUVEC (Fig. 2a, b). In addition, the tube formation assay demonstrated that NALA treatment significantly promoted endothelial network formation of TA-HUVEC (Fig. 2c). Of note, these pro-angiogenic effects were attenuated by the co-stimulation with A-485 (Fig. 2a–c), suggesting that lactate-mediated histone lactylation promoted angiogenesis of TA-HUVEC. Western blot analysis showed that NALA robustly increased the level of histone lactylation, in parallel with the up-regulation of CD31 and VEGFA expressions (Fig. 2d). Notably, A-485 treatment effectively reverses these alteration trend (Fig. 2d), further confirming the involvement of histone lactylation in lactate-induced potentiated angiogenesis of TA-HUVEC. To validate the pro-angiogenic role of lactate in vivo, we established B16F10 xenograft tumors in C57BL/6 mice. When tumors reached 30–50 mm³ (Supplementary Fig. S2a), we administered FX-11, a selective LDHA inhibitor, via intraperitoneal injection. After treatment with FX-11, we observed a significant reduction of intratumoral lactate level (Fig. 2e). In parallel, both tumor volume and tumor weight were prominently suppressed by FX-11 treatment (Fig. 2f, g); these results strongly support that LDHA-mediated lactate production in tumor cells played an oncogenic role. Notably, no significant differences in body weight were observed between treatment groups (Supplementary Fig. S2b). Furthermore, quantitative analysis using 4D Optical Coherence Tomography Structure and Function Evaluation System (4D-OCT) angiography experiments revealed that FX-11 treatment significantly reduces both length and density of blood vessels compared to controls (Fig. 2h and Supplementary Fig. S2c). Concurrent immunofluorescence staining analysis further demonstrated decreased expression of CD31 and H3K18la in CD31^+^ cells following FX-11 treatment (Supplementary Fig. S2d). To further investigate lactate’s role, we utilized intratumoral delivery of NaHCO_3_ nanoparticles (NaHCO_3_ NPs) to specifically neutralize lactate in tumor (Supplementary Fig. S3a) [24]. As was shown, the level of intra-tumoral lactate was prominently reduced, without affecting body weight (Supplementary Fig. S3b, c), while consistently inhibited tumor growth as evidenced by reduced tumor volume and weight (Supplementary Fig. S3d, e). Concurrent immunofluorescence staining analysis revealed that the staining intensity of both CD31 and H3K18la in CD31^+^ cells was decreased in NaHCO_3_ NPs-treated tumors (Supplementary Fig. S3f). Collectively, these results establish lactate as a critical regulator of tumor angiogenesis, with histone lactylation playing a pivotal role in this process.

### ST2 mediates the effect of histone lactylation on angiogenesis

**Fig. 3:**
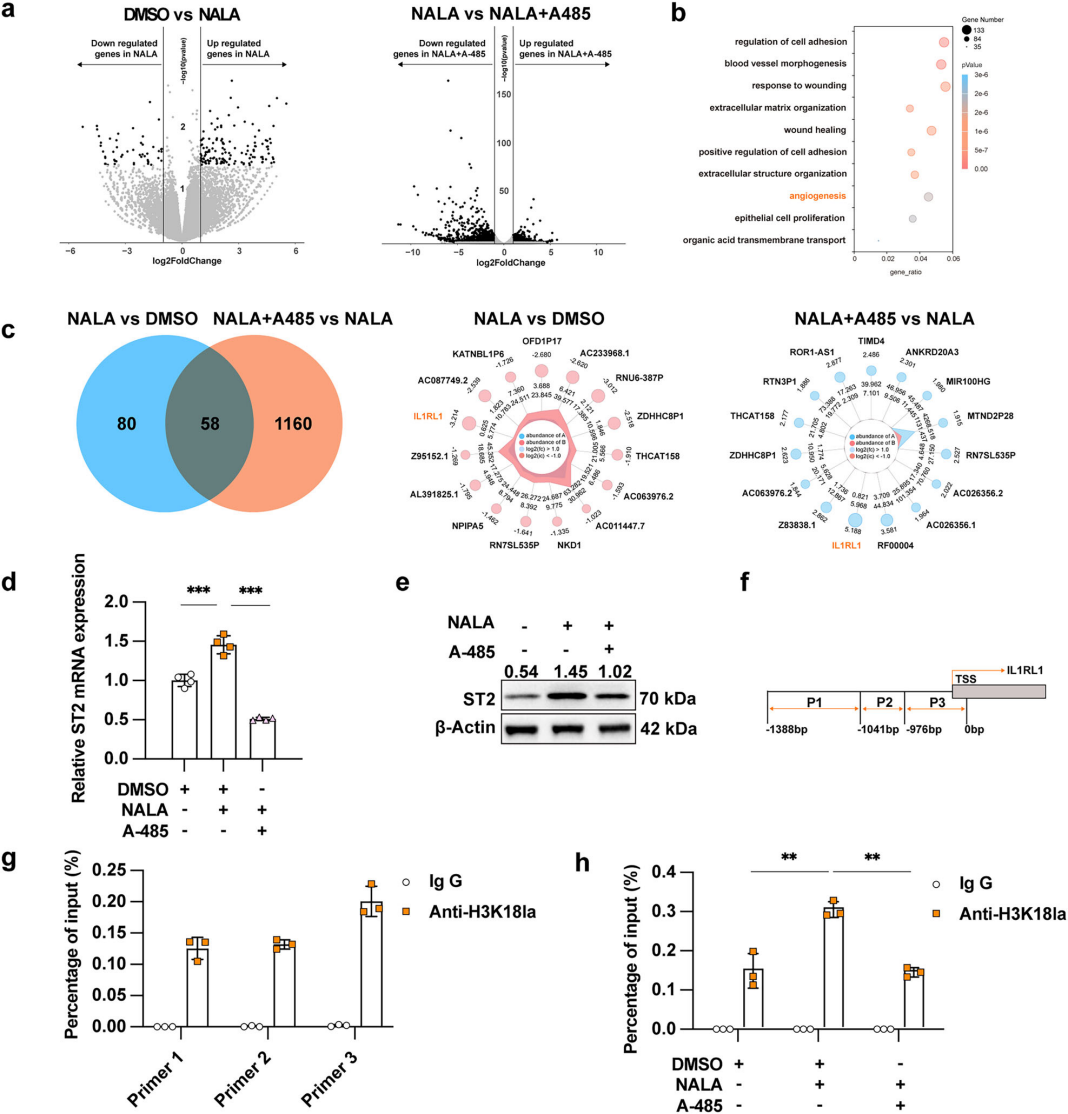
ST2 mediates the effect of histone lactylation on angiogenesis. **a** Differentially-expressed genes in TA-HUVEC between DMSO and NALA-treated group, and between NALA-treated group and NALA plus A-485-treated group according to RNA-sequencing data. **b** GO enrichment analysis of differentially-expressed genes in TA-HUVEC between NALA-treated group and NALA plus A-485-treated group. **c** Venn analysis of differentially-expressed genes between upregulated genes in NALA-treated group compared DMSO group and downregulated genes in NALA plus A-485-treated group compared with NALA-treated group. The radar chart represents the 15 genes with the most significant *p* value in each group, out of the 58 co-expressed genes. **d** Relative mRNA level of ST2 in TA-HUVEC treated with NALA (10 mM) and/or A-485 (10 μM) for 48 h. **e** Immunoblotting analysis of ST2 expression in TA-HUVEC treated with NALA (10 mM) and/or A-485 (10 μM) for 48 h. **f** Schematic view of primers in the promoter region of *ST2*. **g** ChIP analysis of H3K18la enrichment at the *ST2* promoter in TA-HUVEC. **h** ChIP analysis of H3K18la enrichment at the *ST2* promoter in TA-HUVEC treated with NALA (10 mM) or A-485 (10 μM) for 48 h. *P* value was calculated by one-way ANOVA followed by Tukey’s multiple comparisons test, mean ± SEM, *P < 0.05, **P < 0.01, ***P < 0.001.

To investigate the mechanism underlying the effect of lactate and its-dependent histone lactylation on melanoma angiogenesis, we performed RNA-sequencing analysis to identify differentially-expressed genes (DEGs) in TA-HUVEC under three treatment conditions: (1) control, (2) NaLa-treated, and (3) NaLa plus A-485-treated groups. Comparative transcriptomic analysis revealed 191 significantly regulated genes in response to NALA treatment (138 upregulated and 53 downregulated, *p* value < 0.05 and log_2_Fold Change >1), while histone lactylation inhibition (A-485 treatment) modulated the expression of 1,864 genes (646 upregulated and 1218 downregulated, *p* value < 0.05 and log_2_Fold Change >1) (Fig. 3a). Further analysis between NALA-treated group and NALA plus A-485-treated group showed that DEGs were mostly enriched in angiogenesis pathway according to Gene Ontology (GO) enrichment analysis (Fig. 3b). These findings provide compelling evidence that lactate-induced histone lactylation plays a crucial regulatory role in melanoma angiogenesis.

Tumor cells typically mediate intercellular communication with endothelial cells and modulate angiogenesis via the interaction between ligand-receptor, exemplified by the VEGF-VEGFR signaling axis [25]. To identify potential receptor-mediated mechanisms, we focused on genes encoding protein receptors that were differentially regulated by both NaLa and A-485 treatments. Comparative analysis of DEGs revealed that interleukin-1 receptor-like 1 (IL1RL1), a crucial member of the interleukin-1 receptor family, also known as ST2 [26], exhibited significant upregulation following NALA treatment, and re-repressed after co-treatment with A-485(Fig. 3c). ST2 serves as the sole identified receptor for cytokine IL-33 [26]. Emerging evidence demonstrates that the IL-33/ST2 axis promotes angiogenesis in NK/T cell lymphoma by activating Wnt/β-catenin signaling pathway [27]. Based on these findings, we hypothesized that IL-33/ST2 axis might mediate the effect of lactate and histone lactylation on melanoma angiogenesis. Consistent with our RNA-seq data, both qRT-PCR and immunoblotting analysis confirmed that ST2 expression in TA-HUVEC could be promoted after NALA treatment, and this up-regulation could be reversed by the addition of A-485(Fig. 3d, e). Consistent with these findings, parallel experiments using A375-derived TA-HUVECs yielded similar results (Supplementary Fig. S4a, b). Given that histone lactylation modulates gene transcription through promoter region enrichment, we performed chromatin immunoprecipitation (ChIP) assays to examine the enrichment of H3K18la at the promoter of *ST2* (Fig. 3f). As was shown, the three primers spanning the promoter region all identified prominent enrichment of H3K18la (Fig. 3g). More importantly, the enrichment of H3K18la at the promoter of *ST2* was increased in NALA-treated group and re-pressed in NALA plus A-485-treated group (Fig. 3h). To further investigate this, we performed dual-luciferase reporter assays examining the activation of the full-length ST2 promoter (without any truncation or mutagenesis) after the intervention of lactate level or histone lactylation. Our results demonstrate that lactate treatment significantly enhances the activity of the intact ST2 promoter, while this effect is effectively reversed by A-485 co-treatment (Supplementary Fig. S4c), providing evidence that histone lactylation transcriptionally activates ST2 expression in TA-HUVEC. Notably, immunofluorescence and immunohistochemical analyses revealed predominant ST2 expression in tumor-associated endothelial cells within melanoma tissues (Supplementary Fig. S4d, e).

**Fig. 4:**
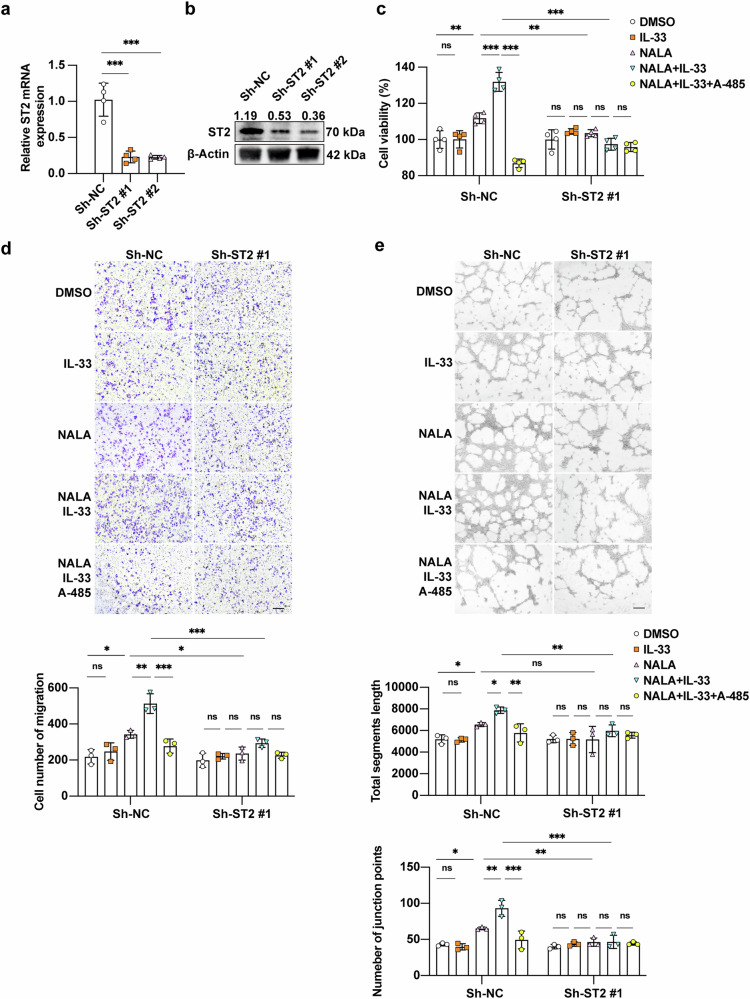
IL-33/ST2 axis promotes angiogenesis in melanoma in vitro. **a** Relative mRNA level of ST2 in TA-HUVEC with or without the knockdown of ST2. **b** Immunoblotting analysis of ST2 expression in TA-HUVEC with or without the knockdown of ST2. **c** Cell viability of TA-HUVEC with or without ST2 knockdown, treated with IL-33 (10 ng/ml), NALA (10 mM) and/or A-485 (10 μM) for 48 h. **d** Cell migration of TA-HUVEC with or without ST2 knockdown, treated with IL-33 (10 ng/ml), NALA (10 mM) and/or A-485 (10 μM) for 48 h. Scale bar, 200 μm. **e** Tube formation of TA-HUVEC with or without ST2 knockdown, treated with IL-33 (10 ng/ml), NALA (10 mM) and/or A-485 (10 μM) for 48 h. Scale bar, 200 μm. *P*-value was calculated by one-way ANOVA followed by Tukey’s multiple comparisons test, mean ± SEM, *P < 0.05, **P < 0.01, ***P < 0.001. ns non-significant.

To functionally characterize ST2, we established stable ST2-knockdown TA-HUVECs using lentiviral transduction, with knockdown efficiency confirmed by both qRT-PCR and Western blot analyses (Fig. 4a, b). While IL-33 mono-treatment did not enhance the proliferation and migration of TA-HUVEC, IL-33 treatment potentiates the effect of NALA on the proliferative and migratory ability of TA-HUVEC. Notably, this synergistic effect was abolished by A-485 co-treatment (Fig. 4c, d). This synergistic effect was abolished by A-485 co-treatment, demonstrating its dependence on histone lactylation. Crucially, genetic ablation of ST2 completely abrogated IL-33’s ability to enhance NALA-mediated angiogenesis (Fig. 4c, d), establishing the essential role of the IL-33/ST2 axis. Consistent with these findings, tube formation assay revealed parallel changes in endothelial network formation across experimental groups (Fig. 4e). Previous report has demonstrated that ST2 promotes angiogenesis in gastric cancer by activating the TRAF6/PI3K/AKT pathway [28]. Therefore, we proposed that ST2 might similarly promote melanoma angiogenesis through AKT activation. Our results demonstrated that lactate plus IL-33 treatment significantly increased phosphor-AKT level, and the knockdown of ST2 could abolish this effect. Moreover, this effect could also be reversed by A-485 treatment (Supplementary Fig. 4f). Hence, the effect of ST2 on melanoma angiogenesis was highly associated with AKT pathway activation. Other downstream pathways might also be implicated in this effect and warrants further investigation. Collectively, these in vitro results demonstrate that ST2 serves as a critical mediator of histone lactylation-driven angiogenesis in melanoma.

### IL-33/ST2 axis promotes angiogenesis in melanoma in vivo

**Fig. 5:**
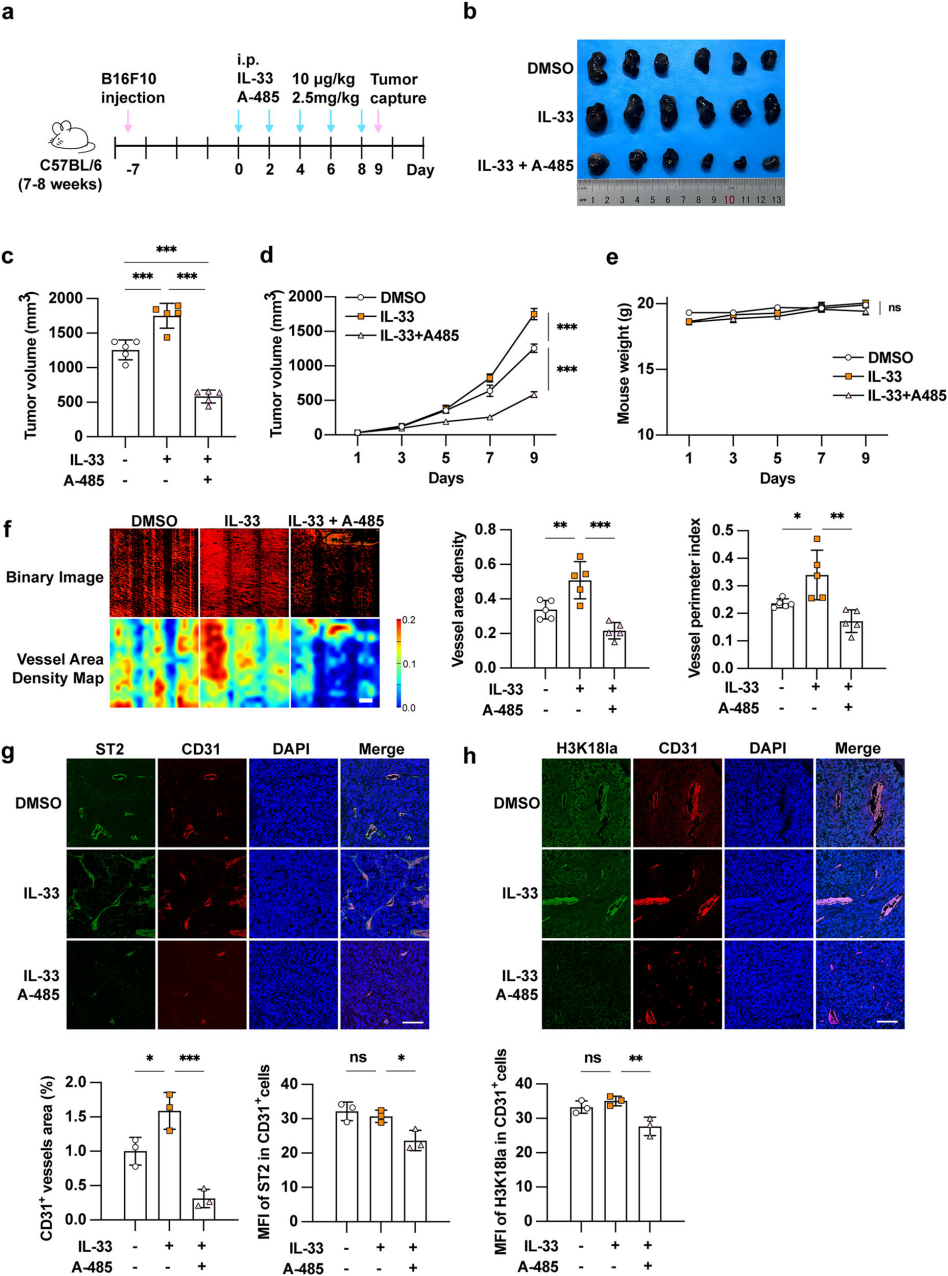
IL-33/ST2 axis promotes angiogenesis in melanoma in vivo. **a** Schematic view of the treatment that C57BL/6 mice burdened with B16F10 melanoma cell tumors with or without mouse IL-33 protein or A-485 treatment. **b** Images of B16F10 melanoma cell xenografts isolated from mice with indicated treatments. Tumor volumes, tumor weights, and mouse weights in each group were calculated and displayed in (**c**, **d**, **e**) (*n* = 5). **f** 4D-OCT intravital imaging of microvasculature in tumor with indicated treatments (*n* = 5, Scale bar, 300 μm). **g**, **h** Immunofluorescence staining of CD31, ST2 and H3K18la in tumor with or without IL-33 mouse protein or A-485 treatment (*n* = 3, Scale bar, 100 μm). *P* value was calculated by one-way ANOVA followed by Tukey’s multiple comparisons test, mean ± SEM, *P < 0.05, **P < 0.01, ***P < 0.001. ns non-significant.

To further investigate the role of the IL-33/ST2 axis in melanoma angiogenesis in vivo, we established B16F10 xenograft tumor models in C57BL/6 mice through subcutaneous inoculation. By the time tumors reached a size of 30–50 mm³, we intraperitoneally injected mice with recombined IL-33 protein or combined with histone lactylation inhibitor A-485 (Fig. 5a). IL-33 treatment significantly increased both tumor weight and volume, while concurrent treatment with A-485 substantially abrogated this alteration trend (Fig. 5b–d). Of note, neither IL-33 treatment nor combined treatment with A-485 affected the weight of mice (Fig. 5e). Quantitative 4D-OCT angiography demonstrated that IL-33 treatment significantly enhanced tumor vascularization, as evidenced by increased vascular area and length parameters, while A-485 co-treatment effectively attenuated these pro-angiogenic effects (Fig. 5f). Immunofluorescence staining analysis showed IL-33 treatment resulted in increased CD31^+^ vessels area and additional A-485 treatment suppressed this up-regulation (Fig. 5g). Although IL-33 exhibited minimal effects on both histone acetylation and ST2 expression in endothelial cells, A-485 treatment significantly suppressed these molecular markers (Fig. 5g, h), likely through inhibition of histone lactylation-mediated ST2 upregulation. Collectively, these findings demonstrated that IL-33/ST2 axis promotes angiogenesis in melanoma in vivo, through a mechanism involving histone lactylation-dependent regulation.

### Lactate and histone lactylation suppress the transformation of HEVs

Beyond angiogenesis, the structural remodeling of endothelial cell plasticity in tumor microenvironment is also detrimental for tumor development [29]. Targeted therapy based on VEGF-VEGFR blockades can simultaneously inhibit angiogenesis and normalize tumor vasculature [30]. High endothelial venules (HEVs) a specialized subtype of post-capillary venules, are anatomically and functionally adapted to facilitate lymphocyte extravasation into lymph nodes and secondary lymphoid organs (SLOs). Furthermore, HEVs can ectopically develop within tumor microenvironments, where they facilitate the formation of tertiary lymphoid structures (TLSs). The presence of TLSs is consistently correlated with favorable clinical outcomes and improved prognosis [31]. Given this clinical significance, we investigated whether lactate and histone lactylation regulate HEV formation in TA-HUVECs. Previous studies have revealed that the maturation and maintenance of HEVs highly relies on lymphotoxin-β receptor (LTβR) signaling, with LTβR agonistic antibody can increase the number of HEVs in vivo [32]. Based on these findings, we employed an LTβR agonistic antibody to induce HEV-like characteristic of TA-HUVEC in vitro. Immunofluorescence staining analysis showed that the staining intensity of MECA-79, a marker of HEV, was prominently up-regulated as the concentration of LTβR agonistic antibody increased (Supplementary Fig. S5a, c), indicating that LTβR activation could induce the HEV-like phenotype in TA-HUVEC in vitro. Notably, we found that NALA treatment significantly attenuated the staining intensity of MECA-79 in LTβR agonistic antibody-treated TA-HUVEC, which could be reversed by co-treatment with A-485 (Supplementary Fig. S5b, d). Thereafter, we examined the selected expressions of two key HEV markers, PNAd and ICAM1, which have been previously reported [33]. Our results demonstrate that NALA treatment significantly suppressed both mRNA and protein expression of PNAd and ICAM1 in TA-HEVs, and this inhibitory effect could be reversed by A-485 (Supplementary Fig. S5e–g). Collectively, these findings establish that lactate-mediated histone lactylation negatively regulates the HEV-forming capacity of tumor-associated endothelial cells.

We next evaluated HEV formation in xenograft tumors following FX-11-mediated lactate inhibition. As was shown, while FX-11 treatment reduced CD31 staining intensity in tumor, the staining of MECA-79 which is localized to CD31^+^endothelial cells, was significantly increased (Supplementary Fig. S5h). Flow cytometry analysis confirmed these findings, demonstrating decreased CD31^+^ endothelial cell populations but increased HEV proportions in FX-11-treated tumors (Supplementary Fig. S6a, b). These in vivo results further support our conclusion that lactate-mediated histone lactylation suppresses tumor-associated HEV formation.

#### The inhibition of LDHA or ST2 increases the efficacy of anti-angiogenic inhibitor in melanoma

**Fig. 6:**
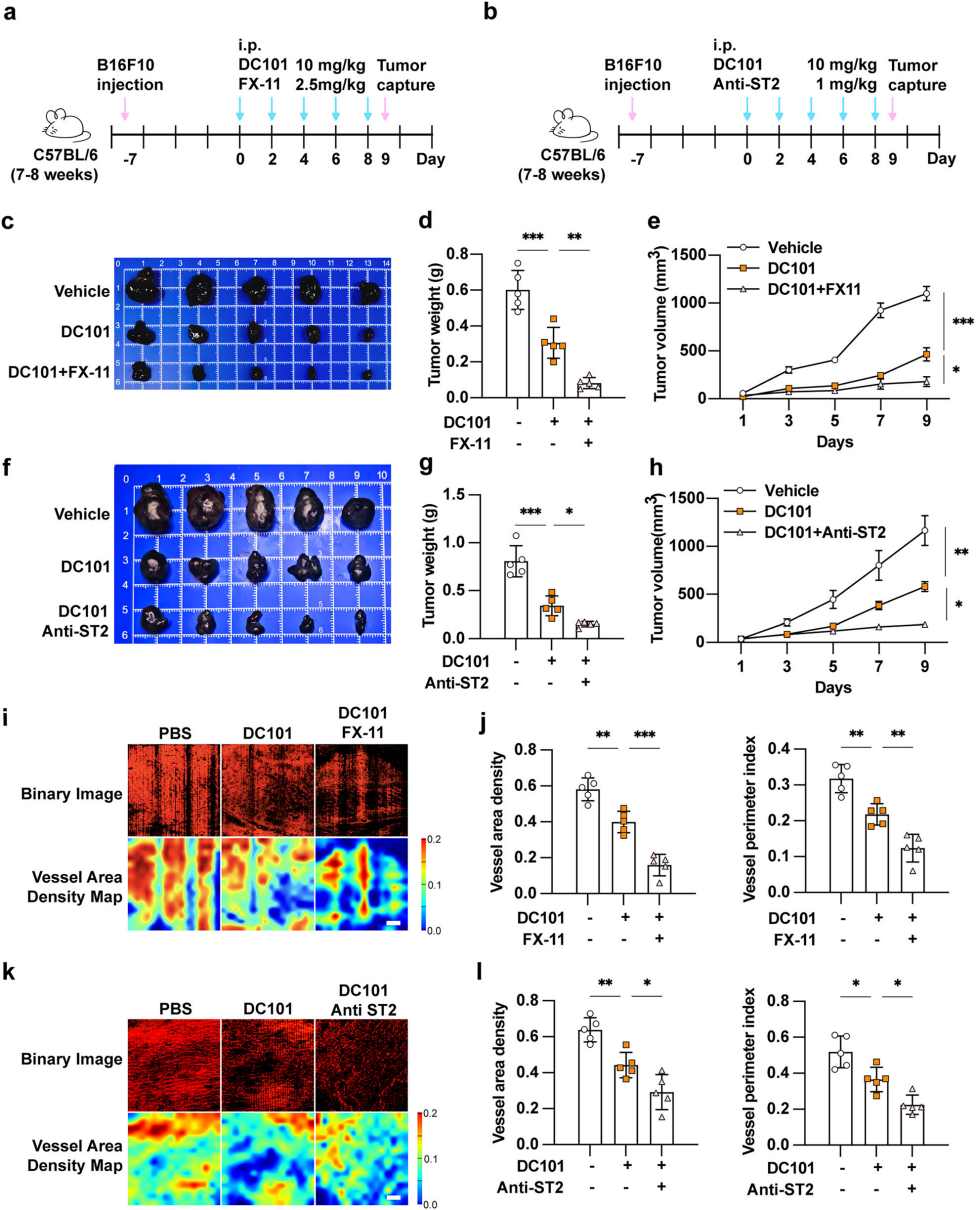
The inhibition of LDHA or ST2 increases the efficacy of anti-angiogenic inhibitor in melanoma. **a** Schematic view of the treatment that C57BL/6 mouse burdened with B16F10 melanoma cell tumors with or without DC101 and/or FX-11 treatment. **b** Schematic view of the treatment that C57BL/6 mouse burdened with B16F10 melanoma cell tumors with or without DC101 and/or anti-ST2 treatment. **c** Images of B16F10 melanoma cell xenografts with indicated treatments isolated from mice. Tumor weights and volumes in each group were calculated and displayed in (**d**, **e**) (*n* = 5). **f** Images of B16F10 melanoma cell xenografts with indicated treatments isolated from mice. Tumor weights and volumes in each group were calculated and displayed in (**g**, **h**) (*n* = 5). **i**, **j** 4D-OCT intravital imaging of microvasculature in tumor with indicated treatments and corresponding quantification (*n* = 5, Scale bar, 300 μm). **k**, **l** 4D-OCT intravital imaging of microvasculature in tumor with indicated treatments and corresponding quantification (*n* = 5, Scale bar, 300 μm). *P* value was calculated by one-way ANOVA followed by Tukey’s multiple comparisons test, mean ± SEM, *P < 0.05, **P < 0.01, ***P < 0.001.

Based on our findings that lactate-mediated histone lactylation upregulates ST2 expression in endothelial cells to activate the pro-angiogenic IL-33/ST2 axis, we next investigated whether targeting this pathway could enhance the therapeutic efficacy of anti-angiogenic treatment. We established B16F10 xenograft tumors in C57BL/6 mice by subcutaneous injection and initiated treatments when tumors reached 30–50 mm³ in volume. (Fig. 6a, b). Monotherapy with the anti-VEGFR2 antibody DC101 significantly inhibited tumor progression, while combination therapy with the LDHA inhibitor FX-11 demonstrated superior antitumor efficacy. (Fig. 6c–e). Similarly, the pharmacological inhibition of ST2 could also increase the efficacy of anti-angiogenic inhibitor DC101 in melanoma (Fig. 6f–h). Of note, these combination treatments showed minimal effects on mice weight (Supplementary Fig. S7a, b). Quantitative 4D-OCT angiography demonstrated that both FX-11 and ST2 inhibition significantly enhanced the anti-angiogenic effects of DC101, as evidenced by marked reductions in vascular length density and perfused vessel area (Fig. 6i–l). Taken together, the combined inhibition of LDHA or ST2 could be promising to increases the efficacy of anti-angiogenic inhibitor in melanoma.

To clinically validate the histone lactylation-ST2 axis in melanoma angiogenesis, we performed immunohistochemical analysis on tumor tissue microarray (TMA) to validate the histone lactylation-ST2 axis in melanoma angiogenesis. In the available tissues consisting of 18 nevus tissues and 74 melanoma tissues, the staining intensity of H3K18la was prominently increased in melanoma instead of nevus (Supplementary Fig. S7c). When stratified by median H3K18la expression, the H3K18la^high^ melanoma group demonstrated markedly increased CD31, VEGFA, and ST2 expression compared to H3K18la^low^ tumors (Supplementary Fig. S7d–f). These clinical findings provide robust translational evidence supporting the functional significance of the histone lactylation-ST2 axis in melanoma vascularization.

## Discussion

In this study, we have systematically elucidated the pivotal role of lactate-mediated histone lactylation in promoting melanoma angiogenesis. First, we found that lactate could enhance the angiogenic capacity of tumor-associated endothelial cells in a histone lactylation-dependent manner. LDHA inhibition or neutralizing the acidic microenvironment in vivo could prominently suppress tumor growth and angiogenesis. Then, transcriptomic analysis identified ST2 as a key mediator of lactate-induced angiogenesis, potentiating endothelial cells’ responsiveness to IL-33 and enhancing their proliferative and migratory capacity. Subsequently, we proved that lactate and histone lactylation also prominently suppressed the characteristics of HEVs in tumor-associated endothelial cells. At last, therapeutic targeting of either LDHA or ST2 sensitized tumors to anti-angiogenic therapy in vivo. These findings establish a novel mechanistic link between tumor metabolism and angiogenesis, wherein lactate-induced histone lactylation transcriptionally activates ST2 expression in endothelial cells, thereby promoting vascular proliferation while inhibiting HEV formation. Our work not only advances the understanding of metabolic regulation in tumor angiogenesis but also provides a compelling rationale for developing combination therapies targeting the lactate-histone lactylation-ST2 axis in melanoma treatment.

While previous studies have predominantly focused on the direct impact of lactate on endothelial cell characteristics, such as the upregulation of VEGF expression in hypoxic environments and the impact on cell proliferative capacity [34]. However, recent investigations demonstrate that lactate interacts with the GPR81 receptor to orchestrate endothelial cell proliferation and migration programs, thereby promoting vascular formation, highlighting lactate’s role as a signaling molecule in angiogenesis, beyond its metabolic role [15, 35, 36]. Building upon these findings, our present study presents a novel epigenetic paradigm of lactate-dependent epigenetic signaling in angiogenesis, highlighting that lactate could regulate the expression of molecules implicated in angiogenic capacity regulation through histone lactylation. Our study provides the first evidence of H3K18 lactylation-mediated transcriptional activation at the IL1RL1/ST2 promoter, establishing a novel epigenetic mechanism for ST2 upregulation in tumor angiogenesis. The vascular parameters (diameter and cross-sectional area) were quantified from 4D-OCT volumetric scans (XYZT coordinates) using a semi-automated segmentation algorithm. Briefly, OCTA (optical coherence tomography angiography) images were generated by analyzing intensity-based decorrelation signals between consecutive B-scans to highlight blood flow. Tumor microenvironment (TME), though heterogeneous across various cancer types, generally comprises immune cells, stromal cells, vasculature, and extracellular matrix (ECM) [37]. Our study demonstrates that lactate-mediated histone lactylation upregulates ST2 expression in vascular endothelial cells, thereby promoting angiogenesis in melanoma. Therefore, it is the ST2 localized in endothelial cells themselves that mediates the effect on angiogenesis. Notably, the ST2 ligand IL-33 exhibits a multi-source expression pattern within the TME. Clinical evidence has shown that intratumoral IL-33 is predominantly localized in cancer-associated fibroblasts (CAFs) in patients with head and neck squamous cell carcinoma (HNSCC) and oral squamous cell carcinoma (OSCC) [38, 39]. Additionally, IL-33 is abundantly produced by B cells in B-cell lymphoma [40], and tumor-draining lymph node macrophages also serve as a major source of IL-33 [41]. The source of IL-33 in melanoma microenvironment needs further investigation. Previous studies have established that IL-33 promotes melanoma vasculogenic mimicry through the IL-33/ST2 axis by upregulating MMP-2/9 via ERK1/2 phosphorylation [42]. Our findings extended this understanding by demonstrating that the IL-33/ST2 axis mediates endothelial cell angiogenesis under the regulation of lactate and histone lactylation. Furthermore, we reveal that lactate’s pro-angiogenic effects encompass not only canonical endothelial cell proliferation but also endothelial cell plasticity— a critical feature whereby endothelial cells dynamically adapt their phenotype and function in response to pathophysiological stimuli, including hypoxia and inflammation [43]. Endothelial cell plasticity serves as a double-edged sword in vascular biology: while essential for physiological tissue repair and vascular remodeling, it also critically contributes to pathological angiogenesis in tumors. Within the tumor microenvironment, the dynamic regulation of endothelial plasticity forms an intricate network that intersects with core cancer hallmarks, including metabolic reprogramming, immune evasion, and therapeutic resistance [29, 33, 44]. Vascular formation, encompassing the transformation of HEVs, represents a significant component of vascular endothelial plasticity. HEVs play a pivotal role in tumor immunology by mediating lymphocyte infiltration and fostering tertiary lymphoid structure formation within the TME [45]. Our findings demonstrate that lactate and histone lactylation impair HEV formation both in vivo and in vitro, revealing a novel mechanism by which lactate modulates vascular plasticity to shape the tumor immune landscape. Given the established therapeutic benefits of tumor-associated HEVs in facilitating the infiltration of lymphocytes into tumor tissues, as well as their involvement in increasing tumor therapy efficacy and improving the prognosis of patients [46, 47], targeting lactate and histone lactylation could be more essential for reconfiguring tumor microenvironment and improving the efficacy of cancer treatment.

Recent advances in anti-angiogenic therapy have demonstrated significant progress in cancer treatment [48, 49]. A particularly promising strategy involves combining anti-angiogenic agents with immune checkpoint inhibitors [50, 51], as anti-angiogenic therapy can enhance immune cell infiltration and potentiate immunotherapy efficacy. For example, the combination of bevacizumab and atezolizumab has remarkable efficacy in treating hepatocellular carcinoma, with a substantial enhancement of the primary endpoint of median progression-free survival when compared the combination therapy to atezolizumab monotherapy [52]. Similarly, the combination of pembrolizumab and axitinib demonstrated superior efficacy and tolerable toxicity in patients with advanced renal cell carcinoma compared to axitinib alone: the 12-month survival rates were 89.9% versus 78.3%, median progression-free survival was 15.1 months versus 11.1 months, and objective response rates were 59.3% versus 35.7% [53]. Furthermore, in a phase 1b study (NCT02298959), 40% of patients with advanced PD-1-resistant melanoma treated with a combination of ziv-afercept and pembrolizumab achieved a partial response or stable disease [54]. Notably, a single-arm clinical trial of Camrelizumab plus Apatinib provided promising antitumor activity with manageable toxicity in patients with advanced mucosal melanoma [55]. While anti-angiogenic therapies have achieved clinical approval and demonstrated efficacy in certain malignancies, overcoming therapeutic resistance and optimizing combination strategies remain critical challenges [56]. Our findings elucidating lactate-mediated regulation of angiogenesis offer new insights into the clinical treatment of melanoma. Specifically, combining LDHA or ST2 inhibitors with existing anti-angiogenic therapies may yield superior antitumor efficacy by simultaneously targeting multiple pathways in the tumor microenvironment.

IL-33, a key immunomodulatory cytokine of the IL-1 family, has garnered increasing attention in cancer immunotherapy as a critical ‘danger signal’ released during cellular stress or damage [57, 58]. It activates various parts of the immune system, particularly through its receptor, ST2. The IL-33/ST2 signaling axis plays a paradoxical role within the TME, where it can either facilitate tumor immune evasion or enhance anti-tumor immune responses [59–61]. Our study reveals a novel epigenetic mechanism by which lactate-mediated histone lactylation regulates ST2 expression in endothelial cells, demonstrating that targeting the IL-33/ST2 axis effectively suppresses lactate-driven angiogenesis in tumors. More importantly, the combined inhibition of either LDHA or ST2 significantly potentiates the efficacy of anti-angiogenic therapy in melanoma, suggesting that targeting the ST2/IL-33 axis could be promising to improve anti-angiogenic treatments in melanoma, as well as in other cancers.

In summary, our study highlights lactate as a key regulator of tumor angiogenesis in melanoma via histone lactylation-mediated activation of the IL-33/ST2 axis in endothelial cells. By establishing how lactate and histone modifications orchestrate angiogenesis, we uncover novel mechanisms by which the TME supports tumor growth and vasculature development. Importantly, we demonstrate that targeting lactate production via LDHA inhibition or blocking ST2 signaling potently enhances the efficacy of anti-angiogenic therapies. These results not only advance our fundamental understanding of tumor vascular biology but also identify promising therapeutic strategies for melanoma and other malignancies characterized by lactate-rich microenvironments.

## Supplementary information

Supplementary Table 1

Supplementary Figure legends

Supplementary Figure S1

Supplementary Figure S2

Supplementary Figure S3

Supplementary Figure S4

Supplementary Figure S5

Supplementary Figure S6

Supplementary Figure S7

Original Data

## Data Availability

The RNA-seq data generated in this study have been deposited in the NCBI GEO database under accession number GSE289136. Other data are available in the manuscript or the supplementary materials.
