# Supplementary material for: Lactate-mediated histone lactylation promotes melanoma angiogenesis via IL-33/ST2 axis
Source: Cell Death Dis. 2025 Oct 6;16(1):701. doi: 10.1038/s41419-025-08023-y (PMC12501017; doi:10.1038/s41419-025-08023-y)
Supplement: Supplementary file 1 — Supplementary Table 1 [file 41419_2025_8023_MOESM1_ESM.docx]

**Supplementary table 1. The primer sequences used for IL1RL1 (ST2) in the qPCR experiment.**

| **Primer name** | **primer sequences** **(5'-3')** |
| --- | --- |
| IL1RL1-F | ATGGGGTTTTGGATCTTAGCAAT |
| IL1RL1-R | CACGGTGTAACTAGGTTTTCCTT |
| siIL1RL1-1F | CACGGUCAAGGAUGAGCAA (dT)(dT) |
| siIL1RL1-1R | UUGCUCAUCCUUGACCGUG (dT)(dT) |
| siIL1RL1-2F | GCACCUCUUGAGUGGUUUA (dT)(dT) |
| siIL1RL1-2R | UAAACCACUCAAGAGGUGC (dT)(dT) |
| siIL1RL1-3F | GAAAGUUCCAGCAAUGACA (dT)(dT) |
| siIL1RL1-3R | UGUCAUUGCUGGAACUUUC (dT)(dT) |
| CHIP-ZNF519-1041F | AATCAGGATGTGCCATCTGCC |
| CHIP-ZNF519-1041R | GGGTGTGATAGGATGGGTATATGAT |
| CHIP-PHOX2B-976F | GTGCCATCTGCCAGAGTCTT |
| CHIP-PHOX2B-976R | GATTACAGGCGTGAGCTACCA |
| CHIP-PHOX2B-1388F | ATAGGCCATCTCGGGCATGT |
| CHIP-PHOX2B-1388R | ATGCCTCGTTGGCAATGTTT |
