# Supplementary material for: Lactate-mediated histone lactylation promotes melanoma angiogenesis via IL-33/ST2 axis
Source: Cell Death Dis. 2025 Oct 6;16(1):701. doi: 10.1038/s41419-025-08023-y (PMC12501017; doi:10.1038/s41419-025-08023-y)
Supplement: Supplementary file 2 — Supplementary Figure legends [file 41419_2025_8023_MOESM2_ESM.docx]

**Supplementary Figure legends**

**Fig. S1**

**a** Relative cell viability of HUVEC and TA-HUVEC (co-culture with A375 cell line) using CCK8 assays at 0, 24, 48, 72 hours. **b** Immunoblotting analysis of CD31 and VEGFA expression in HUVEC and TA-HUVEC (co-culture with A-375 cells). P-value was calculated by unpaired Student's t-test, mean ±SEM, ^*^*P* < 0.05, ^***^*P* < 0.001, ns, non-significant.

**Fig. S2**

**a** Schematic view of the treatment that C57BL/6 mouse burdened B16F10 melanoma cell tumors with or without FX-11 treatment. **b** Mouse weight in each group(n=5). **c** Corresponding quantification of 4D-OCT intravital imaging of microvasculature in tumor with indicated treatments (n=5, Scale bar, 300μm). **d** Immunofluorescence staining of CD31 and H3K18la in tumor with indicated treatments (n=3, Scale bar, 100μm). P-value was calculated by unpaired Student's t-test, mean ±SEM, ^*^*P* < 0.05, ^***^*P* < 0.001, ns, non-significant.

**Fig. S3**

**a** Schematic view of the treatment that C57BL/6 mouse burdened B16F10 melanoma cell tumors with or without NaHCO_3_ NPs treatment. **b** Tumor lactic acid content in each group (mmol/g protein, n=6). **c** Mouse weight in each group(n=5). **d** Images of B16F10 melanoma cell xenografts with indicated treatments isolated from mice. Tumor weights, volumes in each group were calculated and displayed in (**e**) (n=5). **f** Immunofluorescence staining of CD31 and H3K18la in tumor with indicated treatments (n=3, Scale bar, 100μm). P-value was calculated by unpaired Student's t-test, mean ±SEM, ^*^*P* < 0.05, ^**^*P* < 0.01, ^***^*P* < 0.001.

**Fig. S4**

**a** Relative mRNA level of ST2 in TA-HUVEC (co-culture with A375 cell line) treated with NALA (10 mM) and/or A-485 (10 μM) for 48h. **b** Immunoblotting analysis of ST2 expression in TA-HUVEC (co-culture with A375 cell line) treated with NALA (10 mM) and/or A-485 (10 μM) for 48h. **c** Relative luciferase activity of full-length ST2 promoter treated with NALA (10 mM) and/or A-485 (10 μM) for 24h. **d-e** Immunofluorescence and immunohistochemical staining of ST2 in tumor tissues from two melanoma patients (Scale bar, 100μm). **f** Immunoblotting analysis of p-Akt and Akt expression in TA-HUVEC with or without the knockdown of ST2 treated with IL-33(10 ng/ml), NALA (10 mM) and/or A-485 (10 μM) for 48h. P-value was calculated by one-way ANOVA followed by Tukey's multiple comparisons test, mean ±SEM, ^*^*P* < 0.05, ^**^*P* < 0.01.

**Fig. S5**

**a** Immunofluorescence staining of CD31 and MECA-79 in TA-HUVEC treated with various concentrations of anti-AgoLTβR for 48 hours and corresponding quantification (**c**), Scale bar, 100μm. **b** Immunofluorescence staining of CD31 and MECA-79 in TA-HUVEC treated with anti-AgoLTβR (200ng /ml), NALA (10 mM) and/or A-485 (10 μM) for 48 hours and corresponding quantification(**d**), Scale bar, 100μm. **e-f** Relative mRNA level of PANd and ICAM1 in TA-HUVEC treated with anti-AgoLTβR (200ng/ml), NALA (10 mM) and/or A-485 (10 μM) for 48 hours. **g** Immunoblotting analysis of PANd and ICAM1 in TA-HUVEC treated with anti-AgoLTβR (200ng/ml), NALA (10 mM) and/or A-485 (10 μM) for 48 hours. **h** Immunofluorescence staining of CD31 and MECA-79 in tumor from female C57BL/6 mice burdened with B16F10 melanoma cell tumors with or without FX-11 treatment (n=5, Scale bar, 100μm). P-values were calculated using unpaired Student's t-test for comparisons between two groups, and using one-way ANOVA followed by Tukey's multiple comparisons test for comparisons among more than two groups, mean ±SEM, ^*^*P* < 0.05, ^**^*P* < 0.01, ^***^*P* < 0.001.

**Fig. S6**

**a-b** Flow cytometry analysis of CD45^-^CD31^+^ in total tumor cells and MECA-79^+^ in CD45^-^CD31^+^ cells from B16F10 xenografts tumor implanted in C57B6/L mice with or without FX-11 treatment (n=5).

**Fig. S7**

**a** Corresponding quantification of mouse weight in tumor with or without DC101 and/or FX-11 treatment (n=5). **b** Corresponding quantification of mouse weight in tumor with or without DC101 and/or anti-ST2 treatment (n=5). **c** Immunohistochemical analysis of H3K18 expression in melanoma(n=74) and nevus patients (n=18). **d-f** Immunohistochemical analysis of CD31, VEGFA, ST2 expression in low and high lactylation expression groups(n=37). P-value was calculated by unpaired Student's t-test, mean ±SEM, ^*^*P* < 0.05.
