# Supplementary figures and images for: Lactate-mediated histone lactylation promotes melanoma angiogenesis via IL-33/ST2 axis
Source: Cell Death Dis. 2025 Oct 6;16(1):701. doi: 10.1038/s41419-025-08023-y (PMC12501017; doi:10.1038/s41419-025-08023-y)

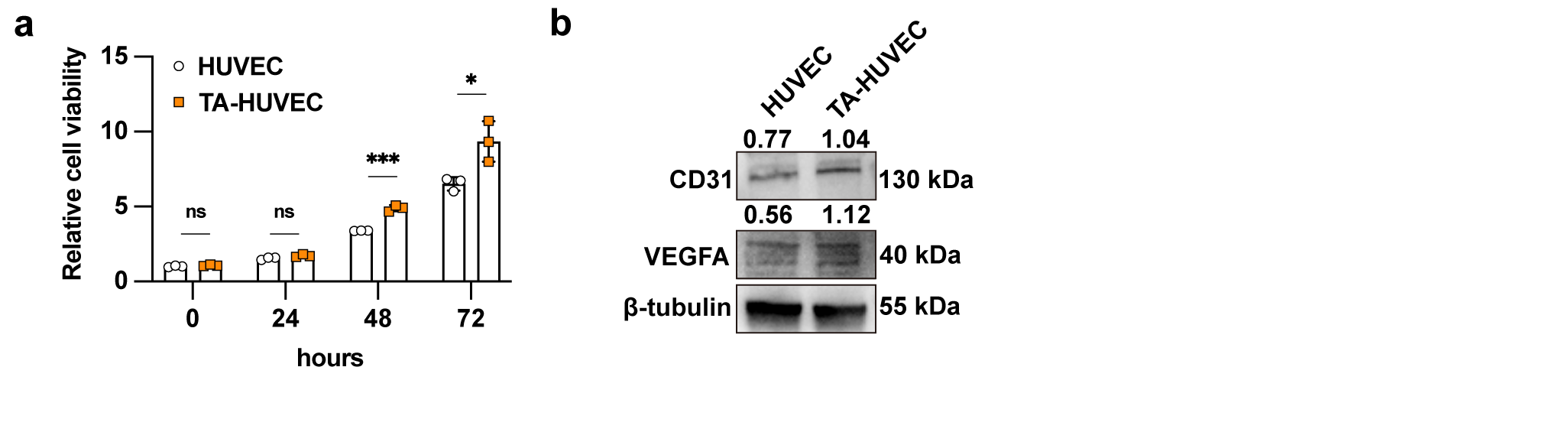

Supplement: Supplementary file 3 — Supplementary Figure S1 [file 41419_2025_8023_MOESM3_ESM.tif]

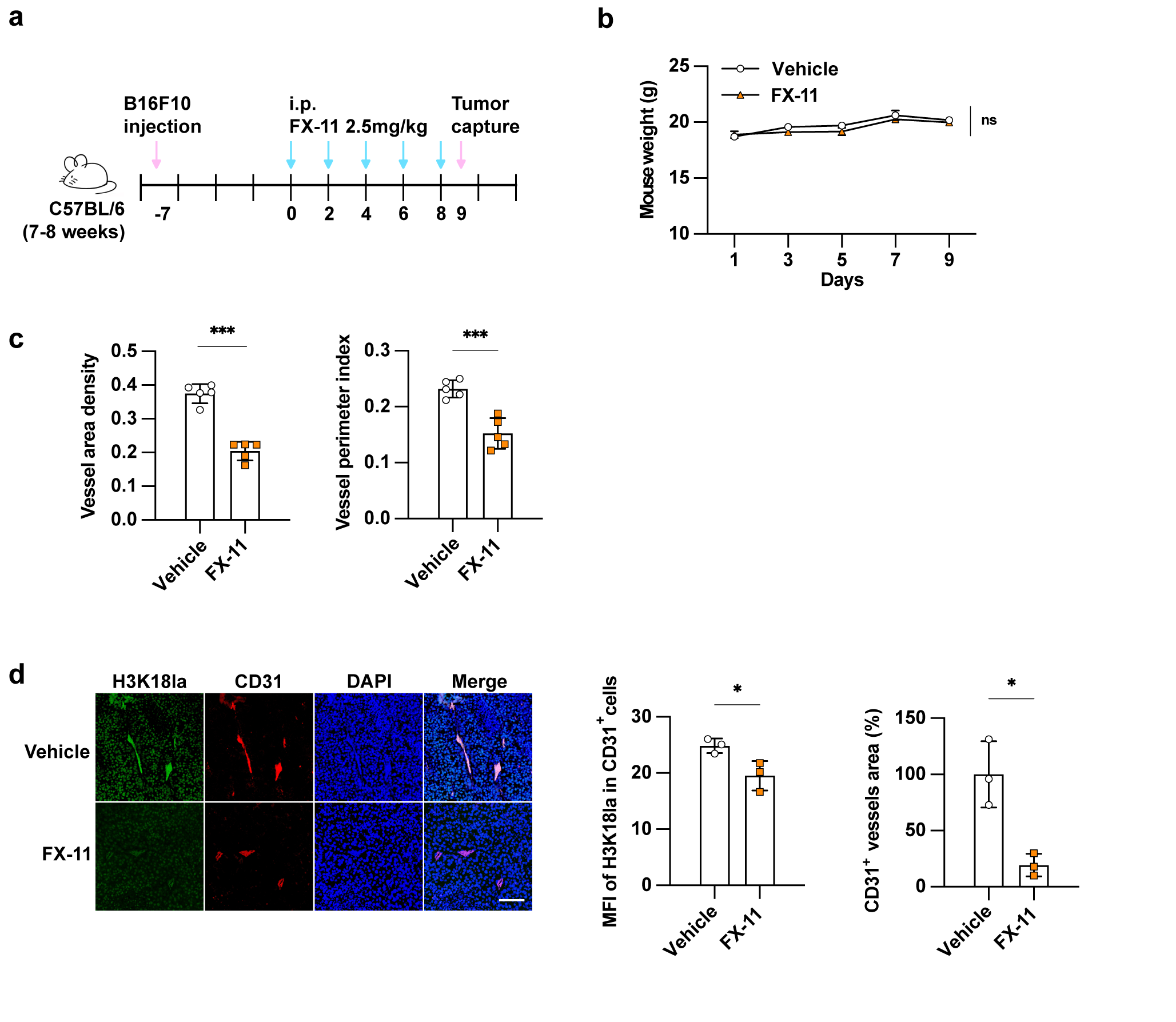

Supplement: Supplementary file 4 — Supplementary Figure S2 [file 41419_2025_8023_MOESM4_ESM.tif]

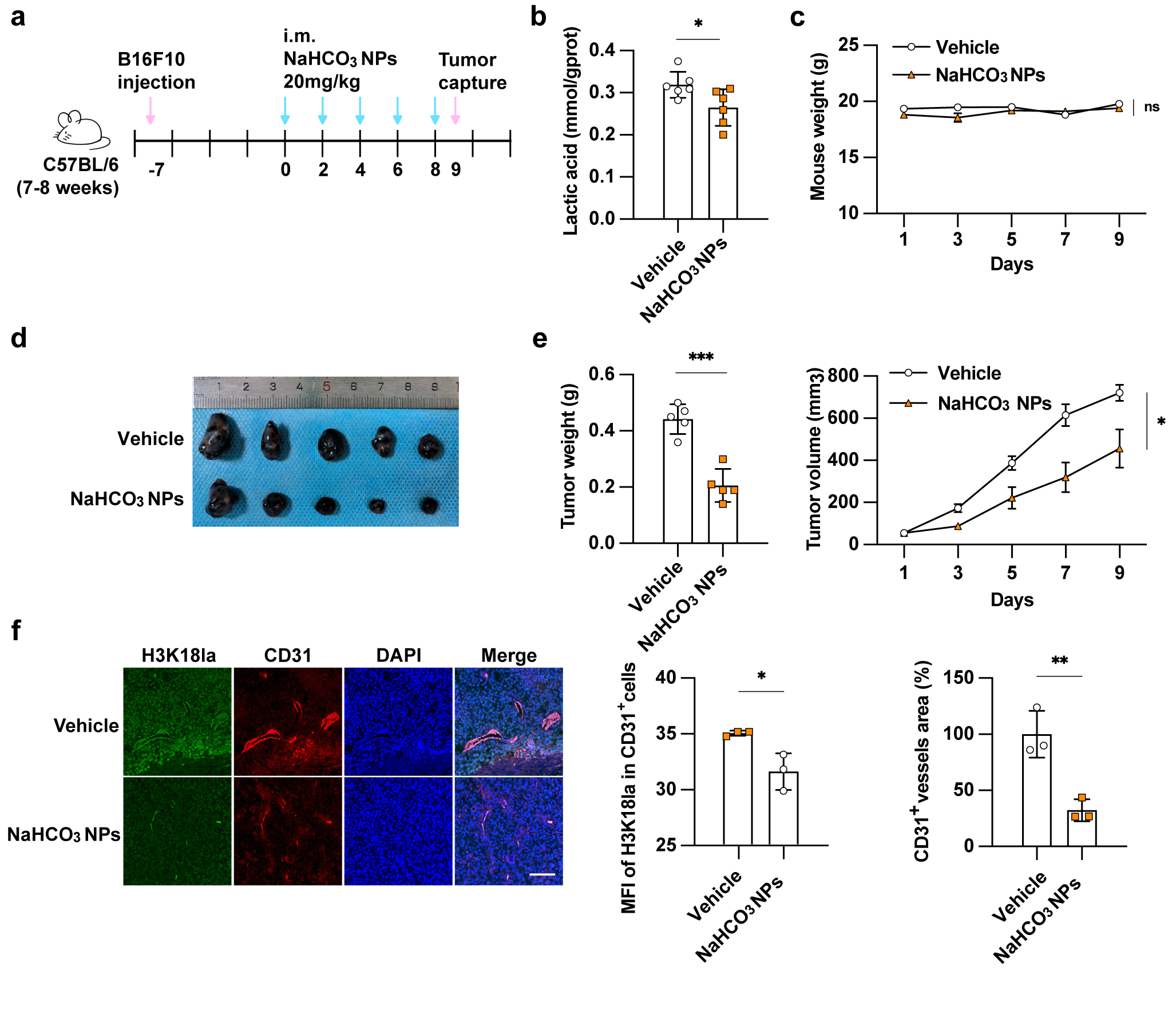

Supplement: Supplementary file 5 — Supplementary Figure S3 [file 41419_2025_8023_MOESM5_ESM.tif]

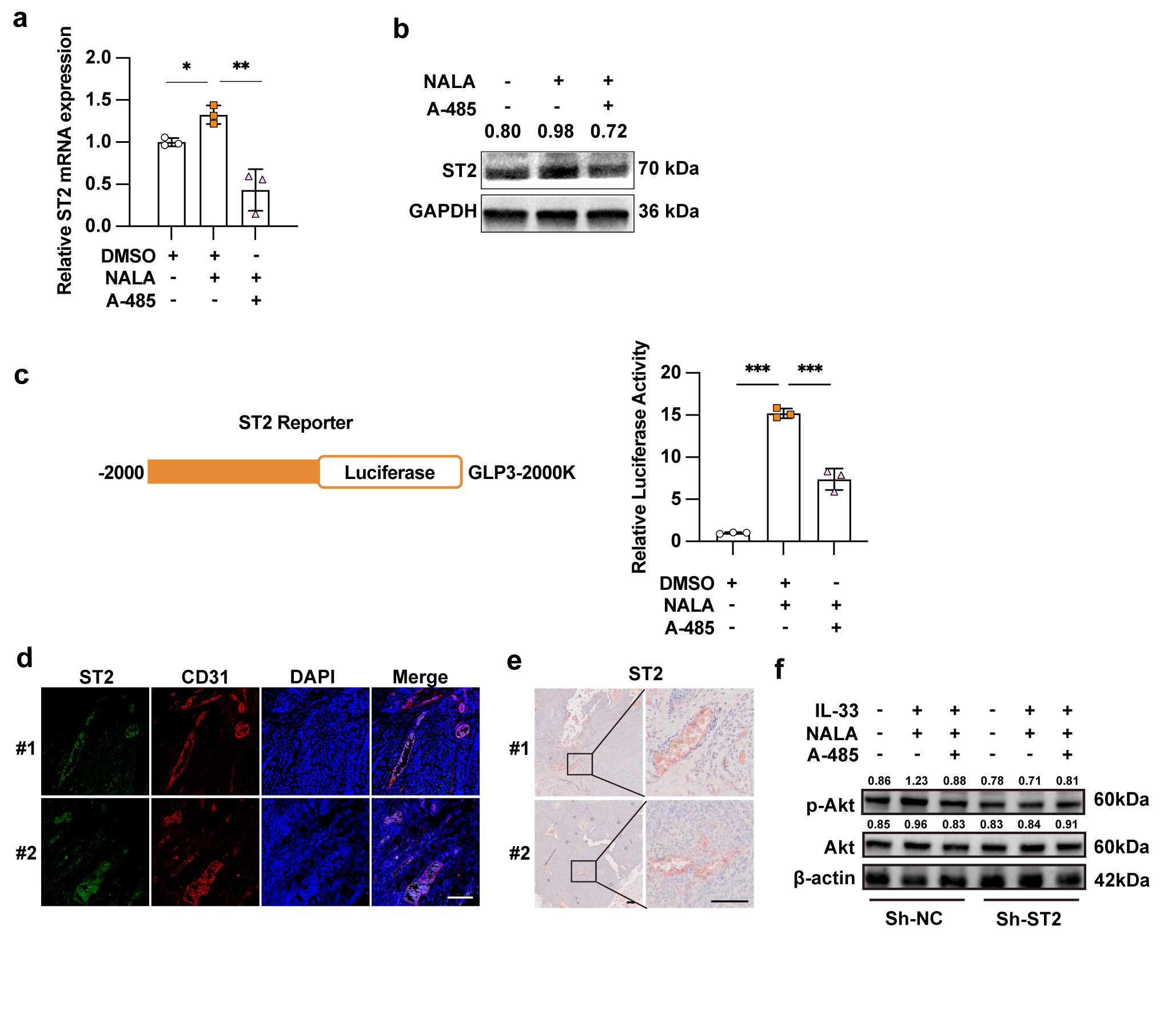

Supplement: Supplementary file 6 — Supplementary Figure S4 [file 41419_2025_8023_MOESM6_ESM.tif]

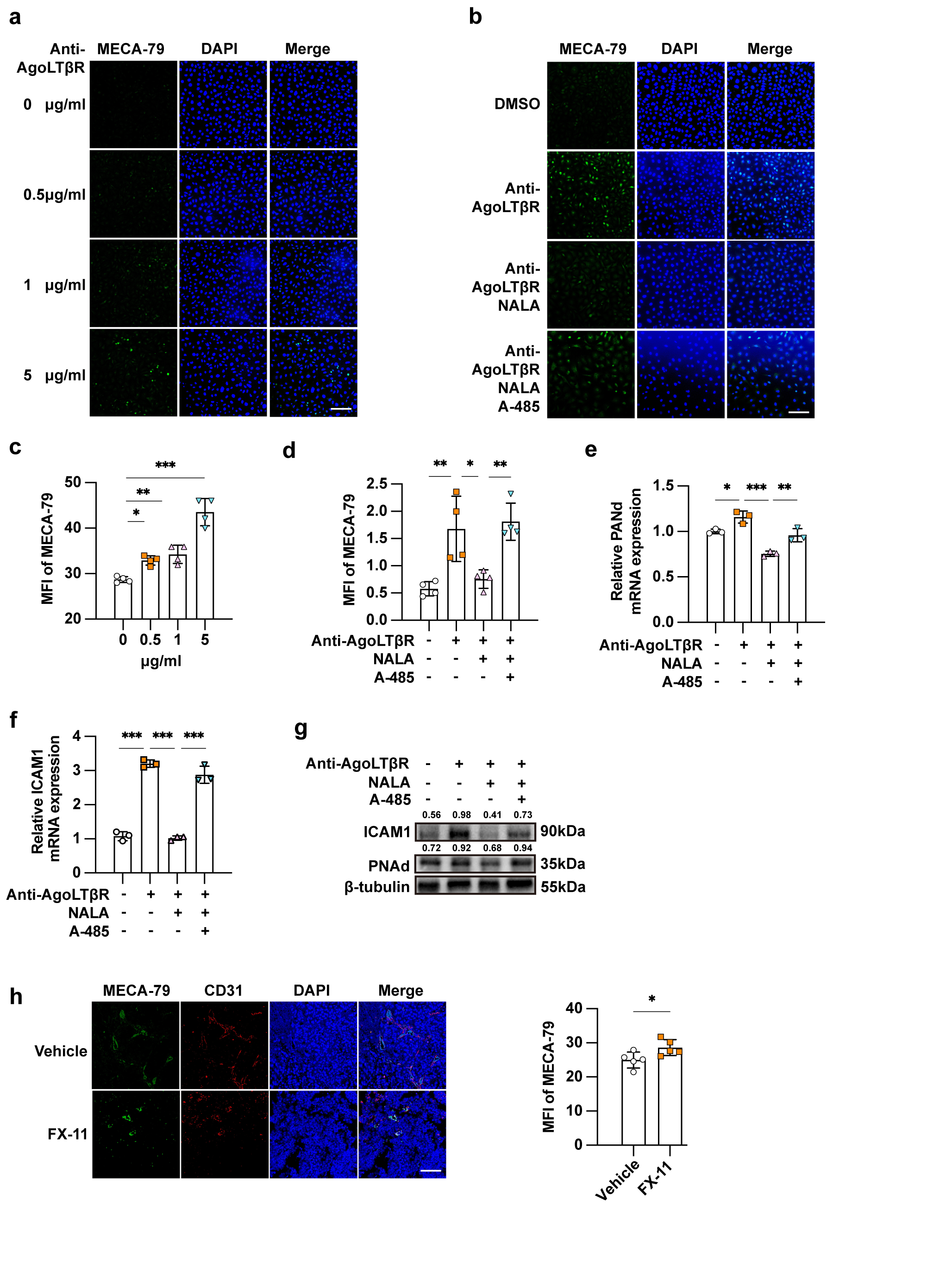

Supplement: Supplementary file 7 — Supplementary Figure S5 [file 41419_2025_8023_MOESM7_ESM.tif]

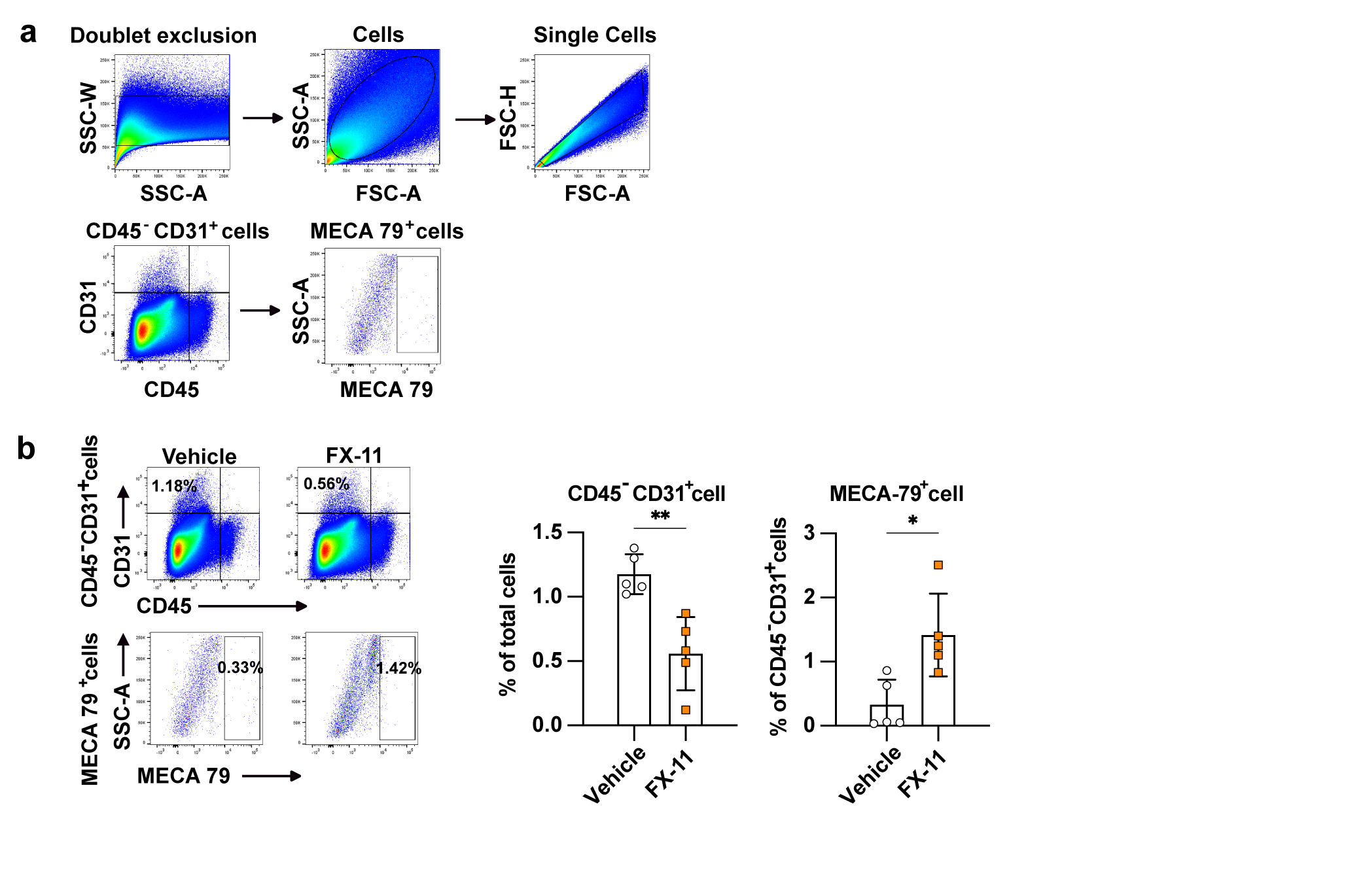

Supplement: Supplementary file 8 — Supplementary Figure S6 [file 41419_2025_8023_MOESM8_ESM.tif]

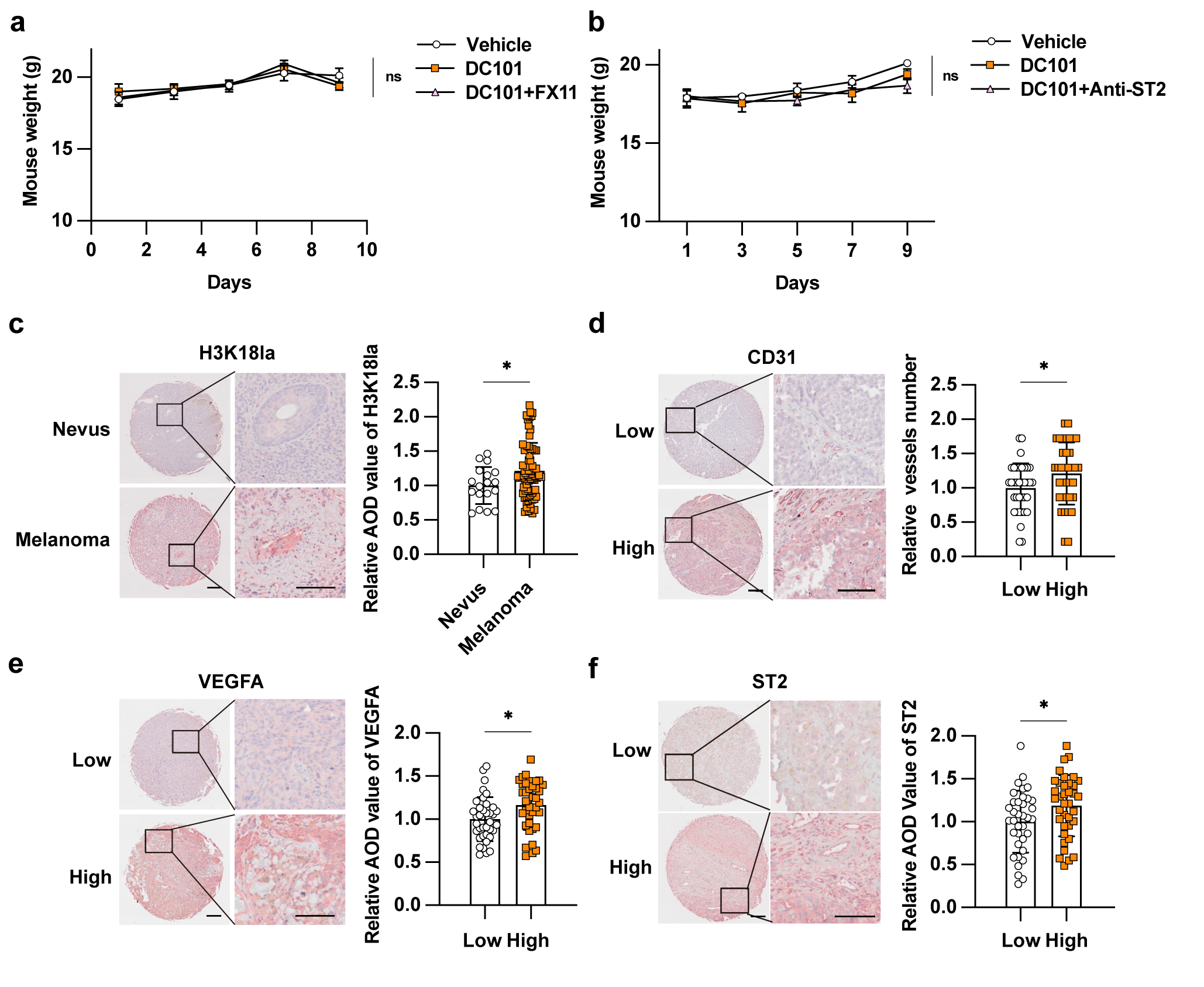

Supplement: Supplementary file 9 — Supplementary Figure S7 [file 41419_2025_8023_MOESM9_ESM.tif]

Original and uncropped films of Western blots

**Fig.1f**

**
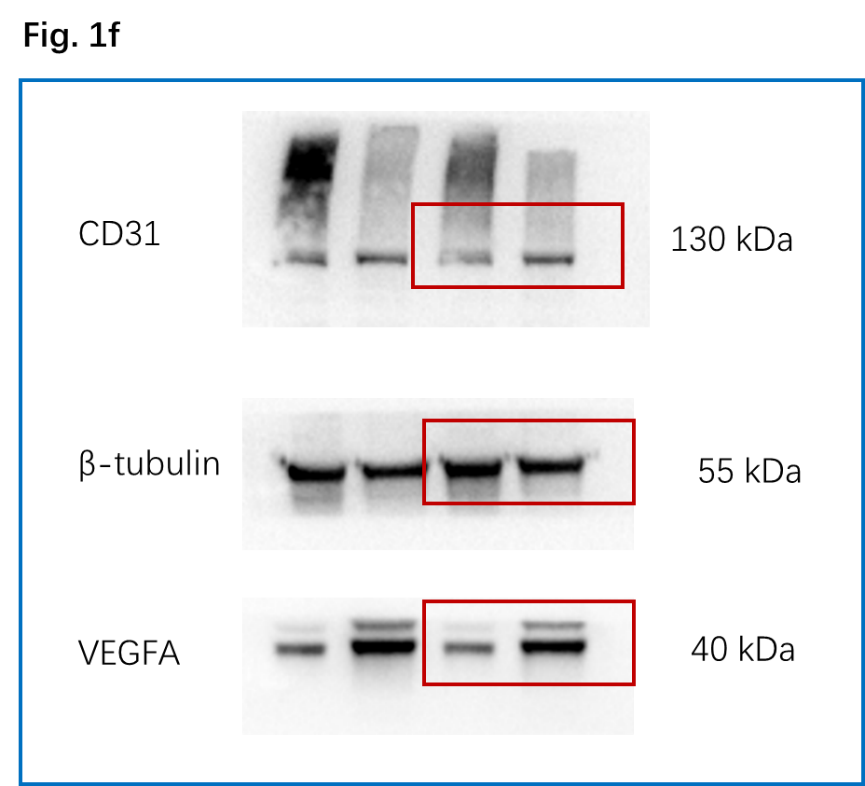
**

**Fig.2d**

**
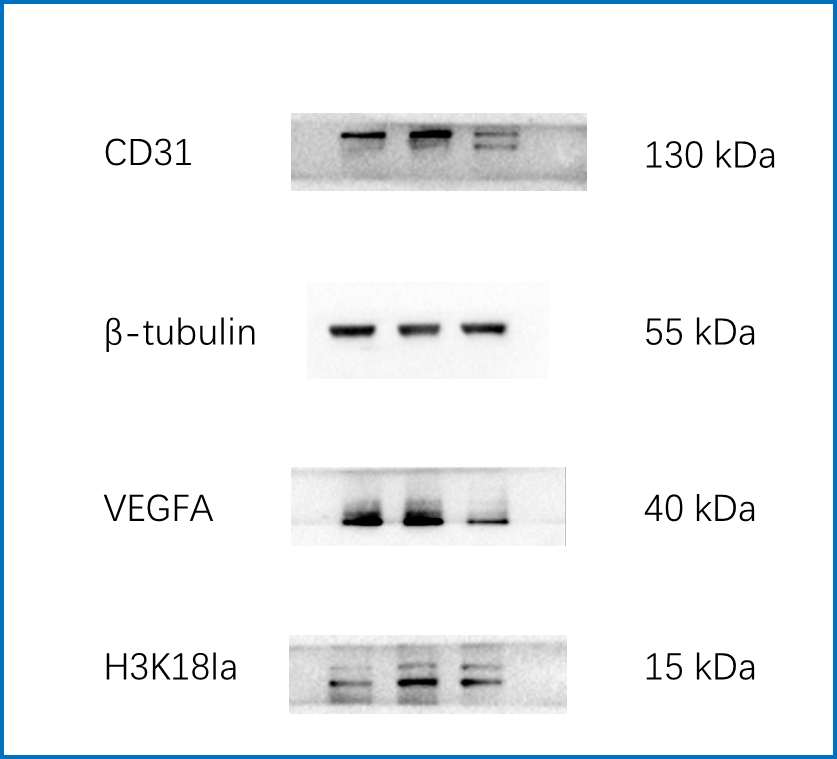
**

**Fig.3e**


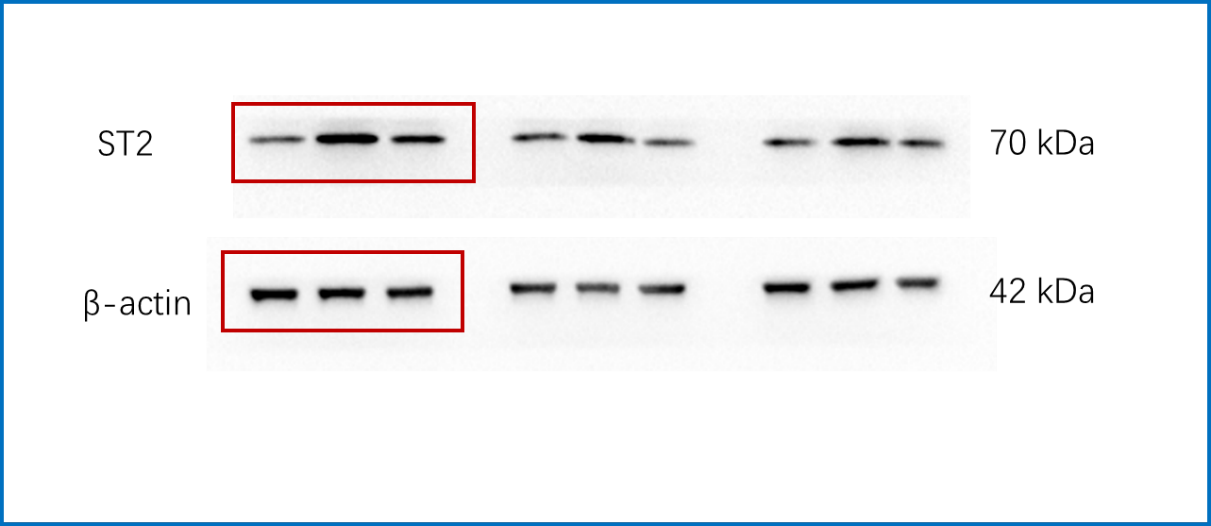


**Fig.4b**


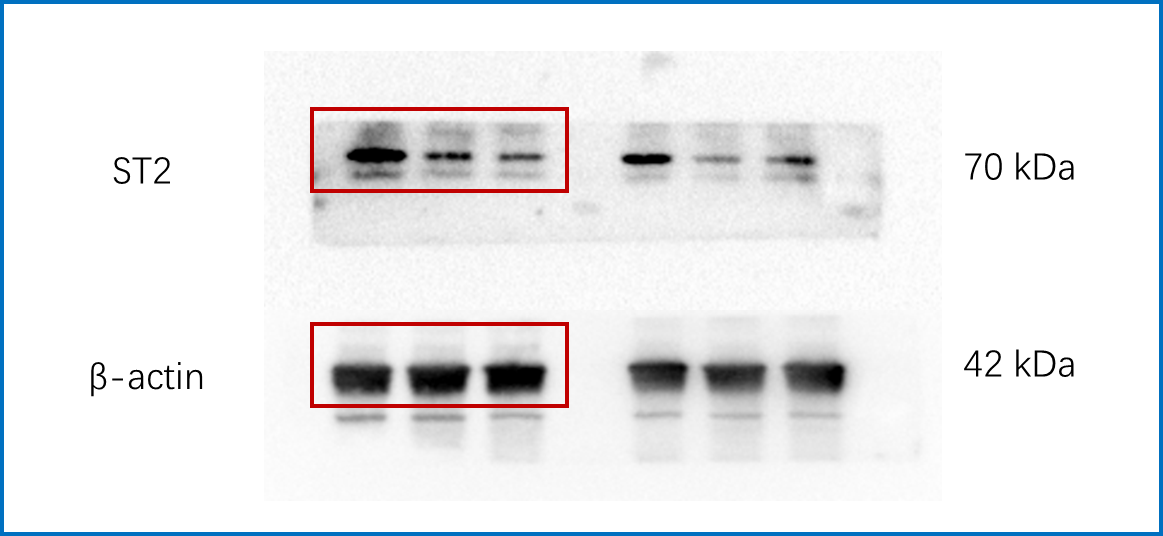


**Fig.s1b**


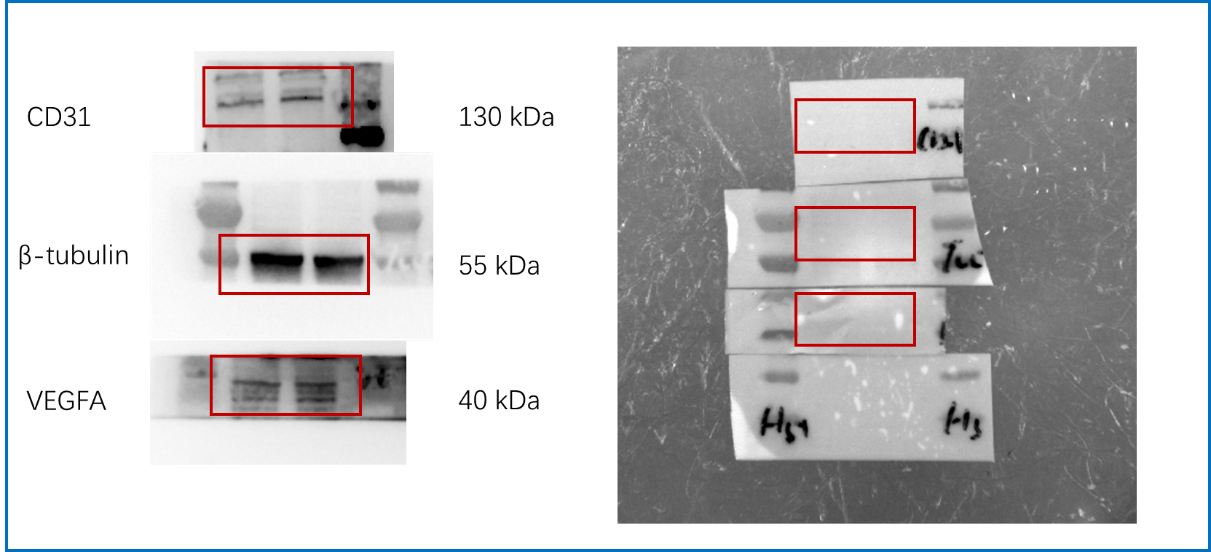


**Fig. s4b**


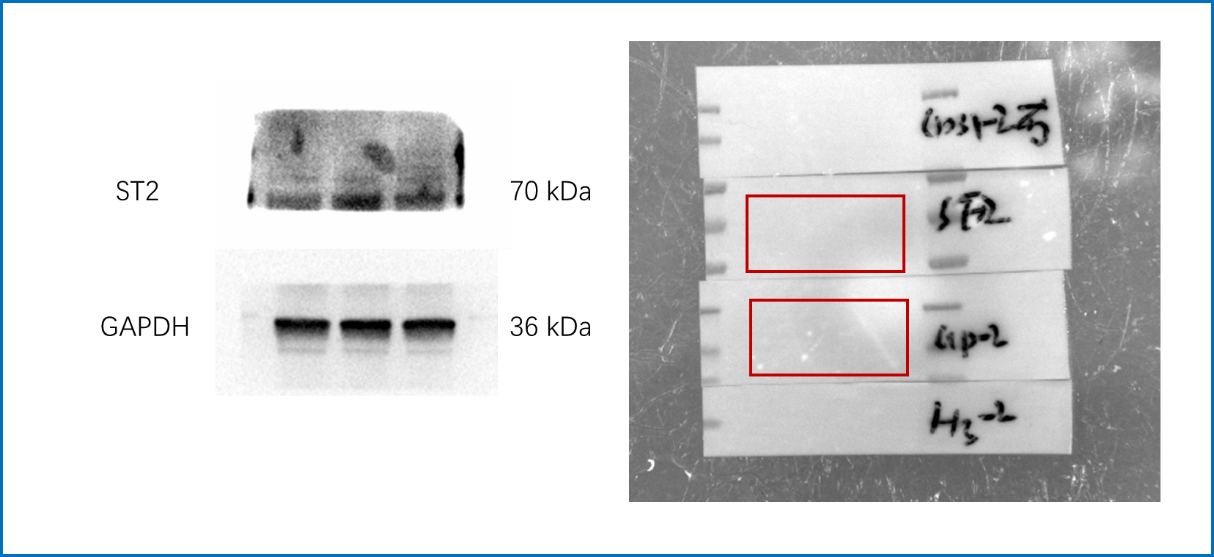


**Fig. s4f**


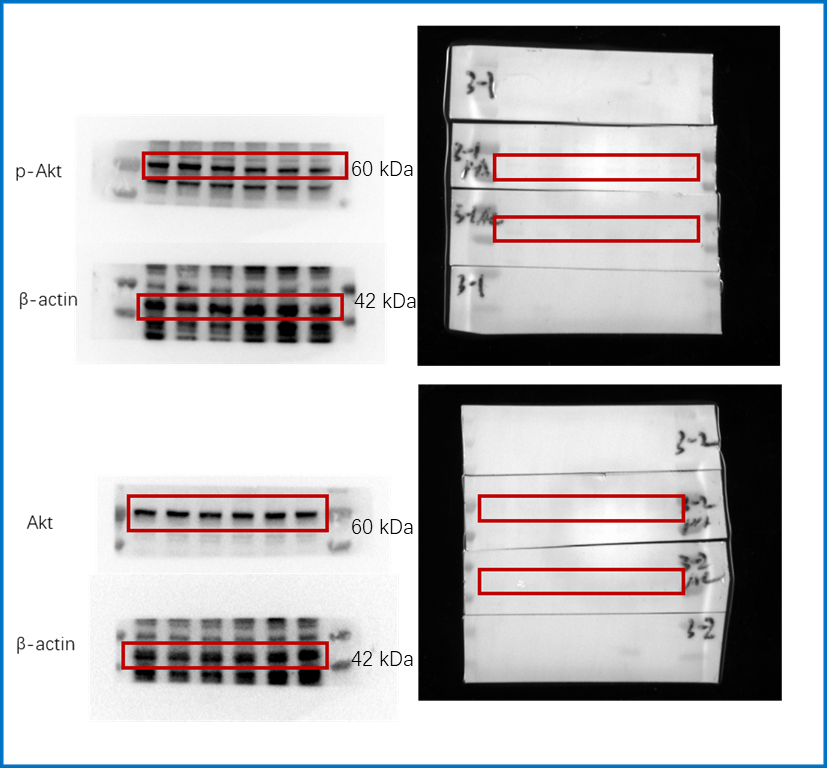


**Fig. s5g**


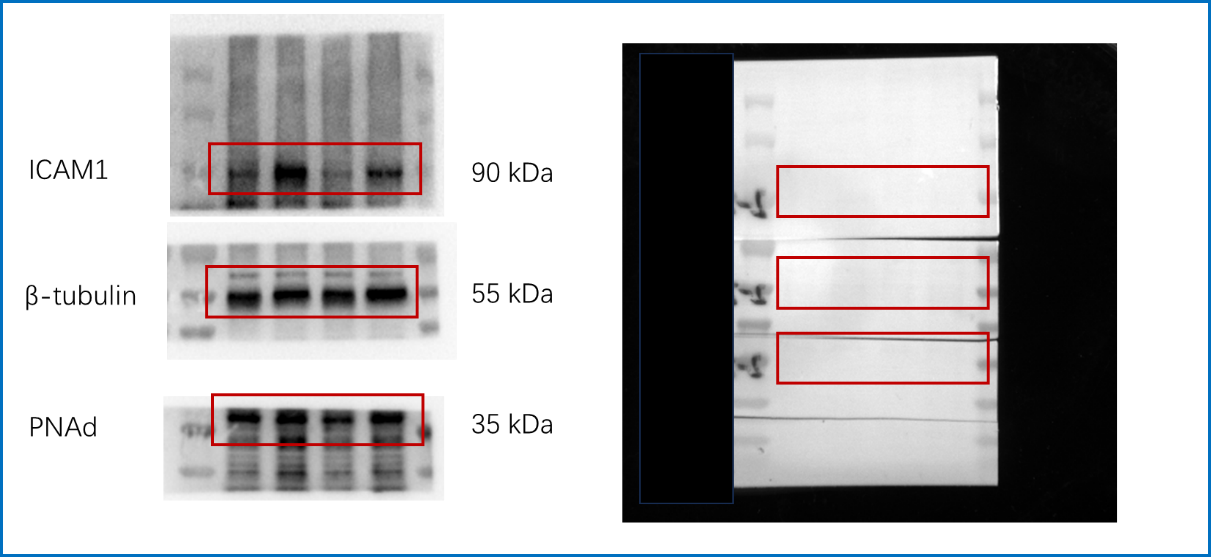

Supplement: Supplementary file 10 — Original Data [file 41419_2025_8023_MOESM10_ESM.docx]
